# Effectiveness of combined versus single circadian interventions in neonatal and pediatric intensive care units: a systematic review with meta-analysis

**DOI:** 10.3389/fneur.2026.1801194

**Published:** 2026-03-25

**Authors:** Constanza C. Corrotea-Maltez, Catalina V. Fernández-Flores, Constanza L. Gutiérrez-Neira, Antonia J. Nirrian-Pérez, Javiera Mansilla-Muñoz, Liliana Bustos-González

**Affiliations:** School of Nursing, Faculty of Medicine, Universidad de Valparaíso, Viña del Mar, Chile

**Keywords:** chronobiology, circadian medicine, circadian rhythms, evaluation of the efficacy-effectiveness of interventions, neonatal intensive care unit, pediatric intensive care unit

## Abstract

**Introduction:**

Admission to neonatal or pediatric intensive care units exposes vulnerable patients to confounding environmental cues, disrupting circadian rhythms and potentially compromising physiological stability. While various circadian interventions exist, ranging from simple light cycling to complex multicomponent bundles, it remains unclear whether complex bundles offer superior clinical benefits over single-modality interventions. This systematic review evaluated the efficacy of circadian interventions on physiological parameters and sleep primarily in critically ill neonates and children, specifically comparing single versus combined strategies.

**Methods:**

A systematic review and meta-analysis were conducted following PRISMA 2020 guidelines. We searched five major electronic databases: PubMed, ScienceDirect, CINAHL, WoS, Scopus and LILACS. Additionally, the Epistemonikos and SciELO databases were screened to identify relevant studies published in languages other than English. The search covered from inception to 2025 and was conducted during October 2025. Risk of bias was assessed using the Cochrane RoB 2 tool and ROBINS-I, while the certainty of evidence was evaluated using the GRADE approach. Data were synthesized using random-effects meta-analysis for physiological outcomes and SWiM guidelines for studies with non-parametric or incompatible data.

**Results:**

Forty-one studies (*N* = 2,548) met the inclusion criteria. Meta-analysis of 16 studies demonstrated that circadian interventions significantly reduced heart rate (SMD -0.70; 95% CI [−1.10, −0.30]; *p* < 0.001) and respiratory rate (SMD −0.75; *p* < 0.001), while improving oxygen saturation (SMD + 1.33; *p* < 0.001) and increasing sleep duration (SMD + 0.92; *p* < 0.001). Narrative synthesis of 16 additional studies confirmed these findings, with 81.2% reporting positive outcomes. Importantly, subgroup analysis revealed no statistically significant difference between single and multicomponent interventions across any outcome (*p* > 0.05). The certainty of evidence was graded as low due to the inherent inability to blind environmental interventions and statistical heterogeneity.

**Conclusion:**

Circadian interventions are effective strategies for modulating physiological parameters and improving sleep quality in the NICU. Single interventions, such as cycled lighting, yielded similar outcomes to complex multicomponent bundles, but due to the lack of pediatric research, our results should be interpreted with caution. Future research should prioritize primary research in pediatric settings to deepen our understanding about circadian interventions.

**Systematic review registration:**

https://doi.org/10.17605/OSF.IO/8SPZM, Identifier: CRD4201091137.

## 1 Introduction

During early life, sleep constitutes a dynamic, active biological process that is fundamental to the maturation of the central nervous system (1, 2). During infancy, the brain undergoes rapid structural organization, which includes synaptogenesis, synaptic pruning, and myelination processes that are inextricably linked to the preservation of sleep architecture and circadian rhythmicity (3–7). Unlike adults, developing infants possess a unique sleep physiology characterized by high proportions of Rapid Eye Movement (REM) and Slow-Wave Sleep (SWS), which facilitate critical functions such as memory consolidation, sensory processing, and emotional regulation (8, 9). Consequently, the integrity of these cycles is a prerequisite for proper neurodevelopment; conversely, sleep fragmentation induces a state of non-restorative sleep that triggers neuroinflammation and has been linked to adverse long-term cognitive and behavioral trajectories (9, 10).

Physiologically, the human circadian system is organized as a hierarchical network governed by the Suprachiasmatic Nucleus (SCN) of the hypothalamus. As the central pacemaker, the SCN orchestrates internal timing by synchronizing peripheral clocks located in nearly every tissue and organ system, including the liver, heart, and immune cells (11). This internal alignment ensures that diverse physiological processes ranging from metabolic flux to hormone secretion, occur in a coherent temporal order (12). The formation of this complex system is a sequential process that begins long before birth. During gestation, the fetal SCN is structurally present but functionally dependent on maternal signals. Among these, the transplacental rhythms of melatonin, cortisol, and body temperature are highly relevant to synchronize its molecular clockwork (6, 7). Birth marks a critical biological transition: the developing brain must switch from this maternal regulation to autonomous rhythmicity, a process that requires exposure to high-amplitude environmental *zeitgebers* (time givers), particularly robust light–dark cycles and predictable social routines (6, 13). These external cues are not merely triggers for sleep; they are the essential drivers that wire the SCN to the rest of the brain, enabling the emergence of stable cortisol rhythms and endogenous melatonin secretion (2, 7). The successful establishment of these robust hormonal rhythms is critical, as it lays the foundation for physiological resilience and optimal health that extends well into adulthood (14, 15).

However, this delicate developmental program is frequently altered by non-normative life events such as hospital admissions due to preterm birth or critical illness. For the preterm infant, birth represents an abrupt, premature severance from the maternal circadian pacemaker. This separation strips the fetus of the transplacental signals that are essential for organizing the SCN, occurring precisely at a time when the brain is most vulnerable and normally shielded by the uterine environment (13). Instead of completing neurodevelopment in the rhythmic protection of the womb, these infants are thrust into the Neonatal or Pediatric Intensive Care Unit (NICU/PICU), environments that fundamentally disrupt this developmental trajectory. Instead of providing the rhythmic cues necessary for SCN maturation, these units present a “sensory mismatch” (8, 13). The immature nervous system is subjected to continuous artificial illumination, excessive auditory stimuli, and invasive 24-h care routines that lack temporal logic (16–18), producing a phenomenon termed circadian disruption or misalignment (12, 19, 20). Recent data confirms that light and noise levels in these units consistently exceed international recommendations. Organization guidelines suggest that noise levels in hospital ward rooms should not exceed a sound pressure level (SPL) of 35 decibels (dB-A) during the day and 30 dB-A at night, recommending that noise in hospital treatment rooms “remain as low as possible” (21). As a consequence, infants lack the environmental conditions required for proper circadian oscillations (10, 13).

To mitigate these adverse effects, the emerging field of Circadian Medicine has proposed specific clinical interventions (22, 23). Crucially, the literature distinguishes these from traditional sleep interventions. While sleep interventions primarily aim to increase the duration or depth of quiescence (often through sedation or environmental silencing) (24), circadian interventions are defined by their intent to restore the timing and amplitude of the biological rhythm. To systematically evaluate these approaches, we established an *a priori* conceptual framework distinguishing between two main categories. The first encompasses primary circadian interventions (e.g., light–dark cycling, chrononutrition), which are specifically designed to entrain the biological clock by mimicking lost maternal or environmental rhythmicity (8, 25). The second category involves supportive sensory modulation strategies (e.g., earmuffs, facilitated tucking, swaddling). While these supportive strategies primarily aim to reduce stress and sensory overload, they may exert indirect circadian benefits by preventing stimulus-induced sleep fragmentation. Together, these approaches aim to consolidate sleep into the biological night and promote active wakefulness during the biological day (26).

Despite this clear physiological rationale, translating these principles into clinical practice presents significant challenges. As highlighted in recent research, the ICU is a complex hospital environment where the implementation of circadian interventions is often hindered by competing clinical priorities and a lack of standardized protocols (27, 28). Consequently, efforts to protect sleep and circadian rhythms are frequently applied inconsistently or in isolation (e.g., using eye masks without controlling noise), failing to address the multisensory nature of the disruption (28).

This implementation gap raises a critical question regarding the complexity of the required solution. Recent systematic reviews suggest that single-component interventions often yield inconsistent results, potentially because they are insufficient to override the multifaceted circadian disruption of the ICU environment (29). Conversely, evidence from adult critical care suggests that combined or multicomponent bundles, which integrate environmental modification with care coordination, may be more effective in reducing delirium and improving sleep quality (30, 31).

Therefore, the objective of this systematic review is to evaluate the comparative effectiveness of combined circadian interventions versus single-component interventions on clinical recovery, sleep quality, and physiological stability in patients admitted to NICUs and PICUs. The results of this review will highlight priority areas for future research and clinical protocols. The goal is to shift current practice toward interventions that not only improve immediate recovery but also preserve the circadian foundations associated with better health outcomes in later life.

## 2 Methods

To write this systematic review, we conducted a literature search following the guidelines of the Preferred Reporting Items for Systematic Reviews and Meta-Analyses 2020 (PRISMA 2020) (32).

### 2.1 Protocol registration

The protocol for this systematic review was registered *a priori* in the International Prospective Register of Systematic Reviews (PROSPERO) (Registration No. CRD4201091137) and posted on the Open Science Framework (OSF)^1^. Additionally, the protocol has been archived as a preprint in medRxiv (33) and was submitted to a peer-reviewed journal, where it currently remains under review. Deviations from the original protocol were implemented prior to study selection to minimize bias and improve the review’s scope. Specifically, language and date restrictions were removed, and eligibility criteria were refined to explicitly include both neonatal (NICU) and pediatric (PICU) intensive care populations.

### 2.2 Research question

This systematic review will answer the focused question: “In neonatal and pediatric patients hospitalized in intensive care units, are combined circadian interventions more effective than single circadian interventions for improving clinical recovery and well-being during the ICU (NICU/PICU) stay?”. The question was developed using the PICO acronym (34) as follows: (1) Population (P): Neonates (preterm or term) and pediatric patients (up to 18 years) admitted to Neonatal or Pediatric Intensive Care Units (NICU/PICU). (2) Interventions (I): Multicomponent circadian-based interventions (strategies combining multiple elements, such as light/dark cycles, noise reduction, and feeding schedules) aimed at enhancing recovery or well-being. (3) Comparators (C): Single-component circadian interventions (e.g., eye masks only, earplugs only) or standard care. (4) Outcomes (O): Clinical recovery or well-being, including length of hospital stay, sleep quality, feeding tolerance, and physiological circadian markers (e.g., cortisol, melatonin).

### 2.3 Eligibility criteria

Study eligibility criteria were established based on the structured PICOS framework (Population, Intervention, Comparator, Outcomes, and Study Design). The inclusion and exclusion criteria were as follows: (1) Population: The review included neonates (preterm or term) and pediatric patients (up to 18 years of age) admitted to intensive care settings, specifically Neonatal Intensive Care Units (NICUs) and Pediatric Intensive Care Units (PICUs). Studies involving mixed ages or mixed units were included only if data for the neonatal/pediatric intensive care population could be extracted or if the study population was predominantly neonatal/pediatric. (2) Interventions: We included studies evaluating circadian interventions aimed at enhancing patient recovery or well-being. These interventions encompassed light exposure regulation, sleep–wake cycle optimization, timing of feeding, and other strategies promoting circadian alignment. (3) Comparators: Eligible studies compared circadian interventions to standard care, no intervention, or alternative circadian approaches (e.g., single vs. multicomponent interventions). (4) Outcomes: Studies were required to report at least one clinical outcome related to patient recovery or well-being. Primary and secondary outcomes of interest included, but were not limited to, hospital length of stay, feeding tolerance, blood glucose regulation, sleep quality, circadian-regulated hormonal levels (e.g., cortisol, melatonin), and physiological parameters (e.g., blood pressure, body temperature, heart rate). (5) Study Design: We included randomized controlled trials (including cluster designs), quasi-experimental studies (e.g., interrupted time series, controlled before–after), and observational designs (cohort, case–control, cross-sectional) evaluating the impact of circadian interventions. (6) Language and Availability: No language or publication date restrictions were applied. Records in languages unfamiliar to the review team were screened using machine translation and, if potentially eligible, assessed by a native or fluent speaker. Only full-text articles (peer-reviewed or preprint) with sufficient methodological detail to support risk-of-bias assessment and data extraction were included. We excluded systematic, scoping, or narrative reviews; qualitative studies lacking quantitative patient-level outcomes; case reports; case series; editorials; and opinion pieces. Studies focusing exclusively on healthcare providers (e.g., nurses or physicians) or adult populations without a distinct neonatal/pediatric subgroup were also excluded. Furthermore, studies focusing solely on environmental factors (e.g., noise or lighting levels) without reporting patient-level clinical or physiological outcomes were not eligible.

### 2.4 Information sources

A comprehensive literature search was conducted, using the following electronic databases: MEDLINE (via PubMed), ScienceDirect (Elsevier), Cumulative Index to Nursing and Allied Health Literature (CINAHL, EBSCO), Cochrane Central Registry of Controlled Trials (CENTRAL, Wiley), Web of Science Core Collection, Scopus, LILACS, SciELO, and Epistemonikos. These databases were selected to provide broad coverage of published studies, encompassing both global and Latin American research contexts. In addition to the electronic database search, we searched the ClinicalTrials.gov and WHO International Clinical Trials Registry Platform (ICTRP) databases to identify ongoing or completed trials. We also screened the reference lists of included studies and performed forward and backward citation chasing to identify additional relevant records. All sources were searched from inception to the date of search, with no language limits applied. The retrieval of all databases was conducted between October 01st-6th, 2025.

### 2.5 Search strategy

The search strategy combined controlled vocabulary from MeSH (Medical Subject Headings) and DeCS (Health Sciences Descriptors) with relevant free-text terms. The core search concepts included: (1) neonatal/pediatric intensive care (e.g., NICU, PICU, intensive care, critical care) and (2) circadian/sleep/chronobiology (e.g., circadian rhythm, chronotherapy, sleep, melatonin, light, noise, day–night). Boolean operators (AND, OR) were applied to refine the results. The strategy was adapted for each database to account for specific indexing systems, syntax, and available filters. The full, database-specific search strings are provided in Supplementary Material 1.

### 2.6 Selection process

**Figure 1:**
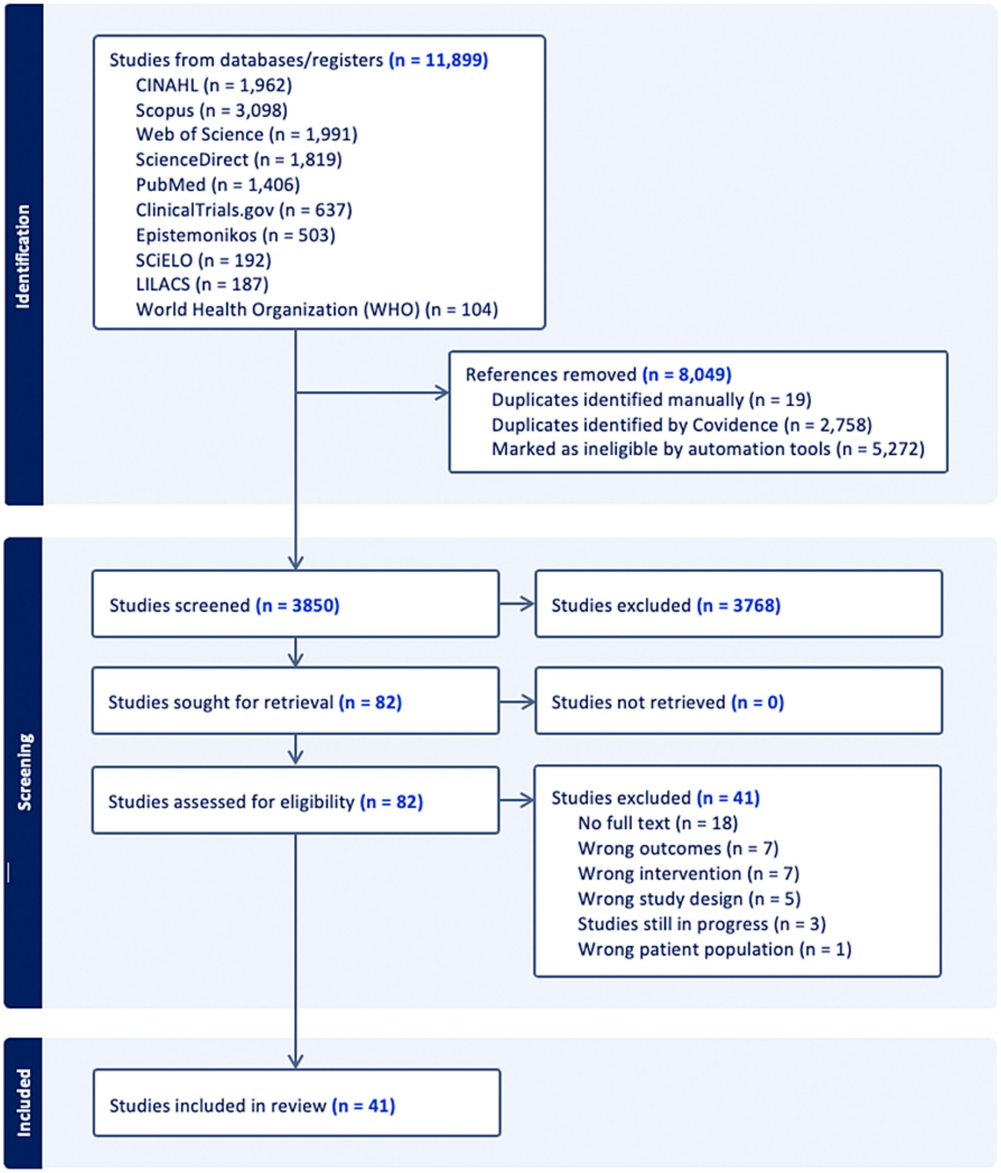
PRISMA 2020 flow diagram illustrating the literature search and study selection process.

All records retrieved from electronic databases were imported into Covidence (Veritas Health Innovation, Melbourne, Australia), a systematic review management platform, which was used to for initial duplicate detection and removal and streamline the screening process as recommended in the Cochrane Handbook for Systematic Reviews of Interventions (35). The study selection was conducted in three stages (1) title screening, (2) abstract screening, and (3) full-text review. Prior to formal screening, a pilot calibration exercise was performed to ensure inter-rater reliability. All reviewers independently screened a random sample of 10 records. As the disagreement rate was less than 10% (indicating >90% concordance), the team proceeded to the formal screening phase. In the first stage, all reviewers independently distributed the data and screened titles and abstracts against the eligibility criteria. In the second stage, full-text articles of potentially relevant records were retrieved and assessed independently as described. Disagreements at any stage were resolved through arbitration by a third reviewer (L. B. G.). Reasons for exclusion at the full-text stage were documented and are reported in the PRISMA flow diagram (Figure 1).

### 2.7 Data collection process

Data extraction was performed using a standardized electronic form developed specifically for this review within the Covidence platform. Prior to full implementation, the form was piloted on a subset of eligible studies to ensure clarity and consistency among the review team. Following this calibration, the included studies were distributed among the reviewers for data extraction. Each study was processed by a single reviewer, and any uncertainties or ambiguities regarding the extracted data were resolved through adjudication by a third reviewer (L. B. G.). Although a protocol was established to contact authors for missing or unclear data, this step was not required as all necessary information was available in the published reports.

### 2.8 Data items

The following variables were extracted from each included study: (1) General characteristics: Study title, lead author, year of publication, country or region, original language, and sponsorship or funding source. (2) Methods: Study design (e.g., Randomized Control Trials (RCT), crossover, retrospective cohort), group configuration, total sample size, follow-up duration, and methodological notes relevant to bias assessment. (3) Population: Inclusion and exclusion criteria, primary clinical diagnosis/condition, age (including gestational age), sex distribution, care unit setting (NICU/PICU), and participant withdrawals. (4) Intervention details: Type of circadian intervention (categorized as single vs. multicomponent), detailed description of the procedure, frequency, intensity (e.g., light lux levels, noise decibels), and total duration of the intervention. (5) Comparators: Description of the control group conditions, such as standard care or alternative environmental settings. (6) Outcomes: Clinical and physiological outcomes categorized as follows: *Clinical Recovery:* Hospital length of stay. *Growth and Nutrition:* Weight gain, head circumference, length, and feeding tolerance. *Sleep:* Quantity (total sleep time) and quality of sleep. *Physiological Parameters:* Heart rate, respiratory rate, oxygen saturation (SpO2), blood pressure, and heart rate variability. *Biomarkers:* Hormonal levels such as cortisol or melatonin.

### 2.9 Risk of bias assessment

The risk of bias was assessed using tools appropriate to each study design. For RCTs, we used the Cochrane Risk of Bias tool version 2 (RoB 2) (36). For the crossover trial included in the review, the specific RoB 2 for crossover trials variant (36) was applied by two independent reviewers (C. C. M. and J. M. M). These tools evaluate bias across five domains: randomization process, deviations from intended interventions, missing outcome data, measurement of the outcome, and selection of the reported result. To manage the review workload, the assessment of RCTs was distributed among the review team, with each study assessed by a single reviewer. Any ambiguities regarding the judgment were resolved through consultation with a third reviewer (L. B. G.). For non-randomized studies (including quasi-experimental and cohort designs), the risk of bias was evaluated using the ROBINS-I tool (Risk Of Bias In Non-randomized Studies – of Interventions) (37). This tool assesses bias across seven domains, encompassing confounding, participant selection, and classification of interventions. Unlike the RCTs, the assessment of non-randomized studies was conducted independently by two reviewers (C. C. M. and L. B. G.) to ensure rigor given the higher inherent risk of bias in these designs. Any discrepancies in the ROBINS-I assessments were resolved through consensus between the two reviewers.

### 2.10 Data synthesis

Data synthesis was conducted in accordance with the Synthesis Without Meta-analysis (SWiM) reporting guidelines (38). A quantitative synthesis (meta-analysis) was performed for outcomes where sufficient data were available and clinical homogeneity was established. For outcomes or studies not suitable for pooling, a structured narrative synthesis was conducted.

#### 2.10.1 Meta-analysis

Studies were considered eligible for pooling if they reported continuous outcomes as mean and standard deviation (SD), or provided sufficient data (e.g., standard error, 95% confidence intervals) to estimate these values. We excluded studies reporting only medians and interquartile ranges (IQR), longitudinal data without summary statistics, or categorical data that could not be converted.

Meta-analyses were performed using a custom script in R statistical software (v4.3.1; R Foundation for Statistical Computing, Vienna, Austria). Effect sizes for continuous outcomes were expressed as standardized mean differences (SMD) with 95% confidence intervals (CI), allowing for the comparison of results across studies using different measurement scales. A random-effects model was applied for all analyses to account for the anticipated methodological and clinical heterogeneity across interventions. Statistical heterogeneity was assessed using the *I^2^* statistic, with an *I^2^* > 50% considered indicative of substantial heterogeneity.

#### 2.10.2 Narrative synthesis

For studies or outcomes that could not be pooled quantitatively, due to insufficient data format (e.g., median/IQR), high heterogeneity (*I^2^* > 85%), or disparate outcome measures, a narrative synthesis was conducted. Results were grouped by outcome domain (e.g., physiological parameters, sleep metrics) and intervention type (single vs. multicomponent). To standardize the reporting of these findings, we prioritized the direction of effect and statistical significance (*p* < 0.05) as reported in the primary studies. Where available, effect estimates were presented alongside their confidence intervals to facilitate interpretation.

#### 2.10.3 Reporting bias assessment

Due to the limited number of studies included in each meta-analysis (*k* < 10 for all outcomes), publication bias could not be formally assessed using funnel plots or Egger’s regression test. According to Cochrane guidelines, tests for funnel plot asymmetry are generally not recommended when there are fewer than 10 studies, as the test power is insufficient to distinguish chance from real asymmetry (35). Consequently, the potential impact of publication bias on the pooled effect sizes remains unknown.

#### 2.10.4 Certainty of evidence

The certainty of the evidence for each outcome was assessed using the GRADE approach (Grading of Recommendations Assessment, Development and Evaluation) (39, 40). The evidence was graded as high, moderate, low, or very low based on five domains: risk of bias, inconsistency (heterogeneity), indirectness, imprecision, and publication bias. For narrative synthesis outcomes, certainty ratings were primarily informed by the consistency of effects and the precision of reported estimates across included studies.

## 3 Results

### 3.1 Study selection

The comprehensive search strategy yielded a total of 11,899 records from electronic databases. After removing 8,049 duplicates using Covidence, 3,850 records remained for title and abstract screening. Of these, 3,768 were excluded for not meeting eligibility criteria.

A total of 82 full-text articles were assessed for eligibility. Following this review, 41 studies were excluded, primarily due to No full text availability (*n* = 18) and Wrong outcome and intervention (*n* = 7, respectively for each reason). Finally, 41 studies met the inclusion criteria and were included in the systematic review. Of these, 28 studies provided sufficient data for quantitative synthesis (meta-analysis). The selection process is detailed in the PRISMA flow diagram (Figure 1).

### 3.2 Study characteristics

A summary of the 41 studies included is presented in Table 1. The publication dates ranged from 1986 to 2025, with a notable increase in research activity in the last 5 years (2020–2025), which accounted for nearly 46% of the included studies (*n* = 19) (41–59). The studies represented a diverse global distribution, with a significant concentration in the Middle East and Asia, being the most represented countries: Iran (*n* = 10) (41, 55, 60–67), Turkey (*n* = 7) (43, 44, 49, 51, 54, 56, 58) and India (*n* = 3) (46, 57, 68). Another important proportion of studies came from the USA (*n* = 7) (47, 48, 50, 69–72) and Brazil (*n* = 3) (53, 73, 74). Finally, European studies (UK, Sweden) were less frequent (*n* = 1, respectively) (75, 76). Thirty-nine studies were published in English and the remaining studies were in Korean (*n* = 2). Regarding the study design, the majority of included studies were Randomized Controlled Trials (RCTs) (*n* = 23) (41, 43–46, 51, 53, 54, 56, 58, 60–64, 66–68, 70–72, 75, 76). Other designs included: crossover trials (*n* = 3) (73, 74, 77); quasi-experimental studies (*n* = 2) (52, 78) and retrospective or prospective cohorts (*n* = 5) (47, 48, 57, 59, 65). The study population consisted predominantly of preterm infants admitted to NICU, with gestational ages ranging typically from 26 to 36 weeks. Specific clinical subgroups included Very Low Birth Weight (VLBW) infants (59). Only two studies focused on pediatric populations outside the neonatal period: one involving infants up to 12 months (48) and one PICU study including patients up to 17 years of age (50). The interventions were categorized into two main groups: The first corresponded to (1) Single-Component Interventions (*n* = 26) (45–49, 53, 54, 57, 58, 60, 61, 63, 64, 68–75, 77–81). These studies evaluated the isolated effect of a specific *zeitgeber*. The most common strategies were lighting modifications like cycled lighting or incubator covers (*n* = 15) (45, 49, 53, 54, 61, 64, 69, 70, 72, 75, 77–81), noise reduction using ear protectors or earmuffs (*n* = 7) (47, 57, 60, 63, 71, 73, 74), and chrononutrition (timed feeding) (58). The other group included (2) Multicomponent Interventions (*n* = 15) (41–44, 50–52, 55, 56, 59, 62, 65–67, 76). These studies utilized “bundles” combining multiple strategies to minimize sensory mismatch (50, 66). Common combinations included light and noise reduction protocols (42–44, 62, 65, 67, 76) often paired with clustered care (52, 55), facilitated tucking (41), or music therapy (59). Regarding the timing and duration of these interventions, significant heterogeneity was observed. Enrollment typically occurred early in the ICU stay, often within the first week of life for preterm infants, though specific eligibility windows varied. The duration of interventions ranged from discrete, short-term applications (e.g., earmuffs applied only during a 2-h quiet time or during specific procedures) to continuous environmental modifications maintained for the entirety of the ICU stay (e.g., cycled lighting continued until discharge). Consequently, the timing of outcome assessments also varied, ranging from immediate post-intervention physiological measurements to cumulative outcomes like hospital length of stay. The most frequently reported outcomes, either by single or combined interventions, were physiological parameters (heart rate, respiratory rate, oxygen saturation) (41–44, 46, 47, 49, 53, 54, 57–61, 63, 67, 71, 76, 79) and sleep metrics (duration, efficiency, and sleep states) (46, 47, 51, 55, 56, 59, 62, 64–66, 68, 73–75, 77, 78, 80). Other key outcomes included weight gain (52, 54, 58, 62, 69, 71, 72, 75, 79, 80), length of hospital stay (LOS) (45, 52, 71, 72, 81), and circadian biomarkers like cortisol and/or melatonin (47, 48, 73, 78).

**Table 1:** Characteristics of the included studies.

Reference	Country	Year	Study design	Mean age (SD)	Sample size	Intervention type	Intervention	Outcomes measured	Outcome impact
Mann et al. (75)	UK	1986	RCT	GA < 36 weeks	41	Single, Primary Circadian	Night/day cycle (light and noise reduction)	Sleep, feeding time, weight gain	Increased sleep; reduced feeding time; 0.5 kg heavier at 3 months (*p* < 0.02)
Miller et al. (69)	USA	1995	Longitudinal interventional study with parallel groups	GA 28 weeks, BW 1100 g	41	Single, Primary Circadian	Cycled lighting	Weight gain, feeding, motor development	Greater weight gain (14% vs. 7.4%/week, *p* < 0.05); earlier oral feeding
Hellström-Westas et al. (77)	Sweden	2001	Crossover	GA 26–34 weeks	18	Single, Primary Circadian	Incubator covers (artificial night)	Sleep states (EEG)	Increased quiet sleep duration; no effect on active sleep
Rivkees et al. (70)	USA	2004	RCT	GA < 32 weeks	38	Single, Primary Circadian	Cycled lighting (LD 12:12)	Rest-activity patterns (actigraphy)	Earlier development of circadian rest-activity patterns
Jung (79)	South Korea	2005	Non-randomized controlled trial	LBW infants	38	Single, Primary Circadian	Cycled lighting	Weight, physiological variables, behavioral states	Improved weight gain and behavioral states
Abou Turk et al. (71)	USA	2009	RCT	GA < 32 weeks, VLBW	34	Single, Supportive Sensory	Silicone earplugs	Weight gain, LOS, physiological stability	No significant difference in weight gain or LOS
Lee et al. (80)	South Korea	2012	Quasi-experimental	GA 32–36 weeks	38	Single, Primary Circadian	Cycled lighting	Sleep time, weight gain	Increased total sleep time; improved weight gain (*p* < 0.05)
Aita et al. (76)	Canada	2013	RCT	GA 26–32 weeks	108	Multicomponent, Primary Circadian	Light and noise reduction program	HR, SpO2	No significant difference in HR or SpO2
Abdeyazdan et al. (60)	Iran	2014	RCT	GA 28–34 weeks	64	Single, Supportive Sensory	Earmuffs for noise reduction	HR, RR, SpO2	Significant reduction in HR and RR; improved SpO2 (*p* < 0.05)
Reyhani et al. (61)	Iran	2014	RCT	GA 30–34 weeks	38	Single, Primary Circadian	Artificial night (incubator cover)	RR, SpO2	Reduced RR; improved SpO2 (*p* < 0.05)
Vásquez-Ruiz et al. (81)	Mexico	2014	Randomized Interventional Study	GA 31–35 weeks	37	Single, Primary Circadian	Artificial night (head cover)	LOS	Reduced length of stay (*p* < 0.05)
Esmaeilizadeh et al. (62)	Iran	2016	RCT	GA < 37 weeks	74	Multicomponent, Primary Circadian	Cycled light and noise reduction	Weight gain, LOS, sleep time	Increased weight gain; reduced LOS; increased sleep time (*p* < 0.05)
Kaneshi et al. (78)	Japan	2016	Quasi-experimental	GA 26–33 weeks	22	Single, Primary Circadian	Red light during night procedures	Melatonin rhythm, sleep, body growth	Earlier melatonin rhythm development; better nocturnal sleep
Araújo et al. (73)	Brazil	2017	Crossover RCT	GA 28–36 weeks	12	Single, Supportive Sensory	Hearing protection (earmuffs)	Salivary cortisol, sleep	Lower cortisol; improved sleep quality
Brandon et al. (72)	USA	2017	RCT	GA < 28 weeks	121	Single, Primary Circadian	Cycled light (early vs. late introduction)	Weight gain, LOS, ventilator days	Early introduction: faster weight gain to 36 weeks PMA
Khalesi et al. (63)	Iran	2017	RCT	GA 28–34 weeks	72	Single, Supportive Sensory	Earmuffs for noise reduction	HR, RR, SpO2	Significant reduction in HR and RR; improved SpO2 (*p* < 0.05)
Valizadeh et al. (64)	Iran	2017	RCT	GA 28–34 weeks	60	Single, Primary Circadian	Light reduction at night	Sleep duration	Increased sleep duration (*p* < 0.05)
Karami and Marofi (65)	Iran	2018	Prospective, randomized, crossover clinical trial	GA 28–34 weeks	66	Multicomponent, Primary Circadian	Noise reduction and light attenuation	Quiet sleep	Increased quiet sleep duration (*p* < 0.05)
Mony et al. (68)	India	2018	RCT	GA < 37 weeks	42	Single, Supportive Sensory	Nesting (environmental stimuli reduction)	Sleep duration, quiet sleep	Increased sleep duration and quiet sleep (*p* < 0.05)
Sato et al. (74)	Brazil	2018	Crossover	Preterm	20	Single, Supportive Sensory	Earmuffs during quiet time	Sleep stages (polysomnography)	No significant difference in sleep stages
Bazregari et al. (66)	Iran	2019	RCT	GA 28–34 weeks	64	Multicomponent, Supportive Sensory	Clustered nursing care	Quiet sleep, active sleep	Increased quiet sleep duration (*p* < 0.05)
Zeraati et al. (67)	Iran	2019	RCT	GA 30–34 weeks	120	Multicomponent, Supportive Sensory	Quiet time protocol	RR	Reduced respiratory rate (*p* < 0.05)
Ezabadi et al. (41)	Iran	2020	RCT	GA 28–34 weeks	40	Multicomponent, Primary Circadian	Artificial night with facilitated tucking	HR, RR, SpO2	Significant reduction in HR and RR; improved SpO2 (*p* < 0.05)
Abdel Hamid et al. (42)	Egypt	2021	Pretest-post test interventional study	GA 28–36 weeks	100	Multicomponent, Primary Circadian	Cycled light and noise reduction	HR, RR, SpO2	Significant reduction in HR and RR; improved SpO2 (*p* < 0.001)
Akarsu and Balci (43)	Turkey	2022	RCT	GA 28–34 weeks	60	Multicomponent, Primary Circadian	Light and noise reducing hat	HR, RR, SpO2	Significant reduction in HR and RR; improved SpO2 (*p* < 0.05)
Íncekar et al. (44)	Turkey	2022	RCT	GA 28–34 weeks	80	Multicomponent, Primary Circadian	Light and noise reduction protocol	HR, SpO2	No significant difference in HR; trend toward improved SpO2
Sánchez-Sánchez et al. (45)	Mexico	2022	RCT	GA 30–35 weeks	73	Single, Primary Circadian	Light–darkness cycle	LOS	Reduced length of stay (*p* < 0.05)
Vadakkan et al. (46)	India	2022	RCT	GA 32–36 weeks	76	Single, Supportive Sensory	Nesting and swaddling	Sleep duration, HR, RR	Increased sleep duration; reduced HR and RR (*p* < 0.05)
Bloch-Salisbury et al. (47)	USA	2023	Prospective Cohort	GA 28–34 weeks	20	Single, Supportive Sensory	Hearing protection device	HRV, sleep efficiency	Improved HRV; trend toward better sleep
Bradford et al. (48)	USA	2023	Retrospective cohort	Infants <12 months	55	Single, Primary Circadian	Exogenous melatonin	Opioid use, pain/sedation scores, adverse events	Reduced opioid use (*p* = 0.049); no adverse events
Çetin and Ekici (49)	Turkey	2023	Repeated measures	GA 37 weeks (median)	64	Single, Primary Circadian	Incubator cover	HR, RR, SpO2, temperature	Improved SpO2 (*p* < 0.05); no significant HR/RR difference
Curley et al. (50)	USA	2024	Two-phase cohort	PICU patients (2 weeks-17 years)	56	Multicomponent, Primary Circadian	RESTORE Resilience Bundle (7 components)	Daytime activity ratio (DARE), melatonin	Improved daytime activity pre-extubation (*p* = 0.04); NS post-extubation
Düken and Yayan (51)	Turkey	2024	RCT	GA 32–37 weeks	120	Multicomponent, Supportive Sensory	Massage therapy and white noise	Sleep duration, sleep efficiency	Increased sleep duration and sleep efficiency in both intervention groups (*p* < 0.05)
Hendy et al. (52)	Egypt	2024	Quasi-experimental	GA 28–34 weeks	53	Multicomponent, Primary Circadian	Clustered care and healing environment (light/noise reduction)	Behavioral outcomes, discharge weight, LOS	Improved behavioral outcomes; higher discharge weight; reduced LOS (*p* < 0.05)
Odebrecht et al. (53)	Brazil	2024	RCT	GA 30–35 weeks	40	Single, Primary Circadian	Light protection equipment (eye masks) at night	Days until discharge, HR variability	Earlier discharge (8 vs. 12 days, *p* = 0.025); greater HR day-night variation
Olgun et al. (54)	Turkey	2024	RCT	GA 28–34 weeks	30	Single, Primary Circadian	Light–dark cycle	Weight gain, HR	Improved weight gain; no significant HR difference
Sabagh and Ghaljaei (55)	Iran	2024	Non-randomized controlled trial	Preterm	62	Multicomponent, Primary Circadian	Quiet time protocol (light/noise reduction + clustered care)	Sleep status (categorical)	58.1% vs. 0% achieved deep sleep (*p* < 0.001)
Suna Dağ et al. (56)	Turkey	2024	RCT	GA 26–32 weeks	99	Multicomponent, Supportive Sensory	White noise and facilitated tucking	Sleep efficiency	Improved sleep efficiency (*p* < 0.05)
Ray et al. (57)	India	2025	Prospective observational, non-randomized, controlled cohort	GA 28–34 weeks	100	Single, Supportive Sensory	Earmuffs for noise reduction	HR, RR, SpO2	Significant reduction in HR and RR; improved SpO2 (*p* < 0.001)
Temizsoy et al. (58)	Turkey	2025	RCT	GA 26–32 weeks	62	Single, Primary Circadian	Chronobiological feeding model	Weight gain	Increased weight gain (*p* < 0.05)
Zhao et al. (59)	China	2025	Retrospective cohort	VLBW infants	100	Multicomponent, Supportive Sensory	Music therapy and multidimensional nursing	HR, RR, SpO2, sleep time	Reduced HR and RR; improved SpO2; increased sleep time (*p* < 0.05)

SD, standard deviation; RCT, randomized control trial; GA, gestational age; LD, light/dark; EEG, electroencephalogram; LOS, length of Stay; HR, heart rate; SpO2, oxygen saturation; RR, respiratory rate; LBW, low birth weight; HRV, heart rate variability; VLBW, very low birth weight.

### 3.3 Risk of bias in studies

To assess the risk of bias in the included studies, we used tools appropriate to each study design. The 23 included RCTs (41, 43–46, 51, 53, 54, 56, 58, 60–64, 66–68, 70–72, 75, 76) were evaluated using the Cochrane RoB 2 tool (36), while the 3 crossover studies were assessed using the specific RoB 2 tool for cross over studies (73, 74, 77). The 15 non-randomized, prospective, retrospective, cohort or quasi-experimental studies (42, 47–50, 52, 55, 57, 59, 65, 69, 78–81) were evaluated using ROBINS-I (37). Among the 23 RCTs, the overall risk of bias was rated as High in 18 studies (78.3%) (41, 43, 44, 46, 51, 54, 56, 58, 60–64, 66–68, 70, 75, 76), Some Concerns in 4 studies (17.3%) (45, 53, 71, 72), and Low in only 1 study (3.8%) (58). The primary driver of this assessment was deviations from intended interventions (Domain 2, 95% rated as High). Due to the nature of environmental interventions (e.g., cycled lighting, noise reduction), it was often impossible to blind participants or care providers, leading to a high risk of performance bias in 84.6% (*n* = 22) of trials (41, 43–46, 51, 53, 54, 56, 60–64, 66–68, 70–72, 75, 76). However, selection bias (Domain 1) was generally well-managed, with 69.5% of studies showing Low risk or Some Concerns (21.7%) regarding the randomization process. Similarly, reporting bias (Domain 5) was low, with 73.9% (*n* = 17) of studies showing no evidence of selective reporting (43–46, 51, 53, 54, 56, 58, 63, 64, 67, 68, 71, 72, 75, 76). In addition to the parallel-group trials, the included 3 crossover trials (73, 74, 77) were similarly classified as High risk due to the inherent performance bias described above.

For the 15 non-randomized studies, the overall risk of bias was rated as Moderate in 9 studies (60.0%) (52, 55, 57, 59, 69, 78–81) and Serious in 6 studies (40.0%) (42, 47–50, 65). No studies were classified as Critical or Low risk. The most prevalent sources of bias were confounding (Domain 1) and measurement of outcomes (Domain 6). Confounding was a moderate issue in 66% (*n* = 10) (47, 49, 52, 55, 57, 69, 78, 79, 81, 82) of studies due to the lack of randomization and insufficient adjustment for variables such as illness severity. Measurement bias was identified as a moderate-to-serious risk in all 9 studies (60%), primarily because behavioral outcomes (e.g., sleep state, crying) were assessed by unblinded observers aware of the intervention status (42, 48, 49, 55, 59, 65, 79–81). Conversely, biases related to participant selection and missing data were minimal. Remarkably, in the domain of classification of interventions (Domain 3), 100% of non-randomized studies were evaluated as low risk. This reflects the unambiguous nature of environmental circadian interventions (e.g., lighting protocols, physical noise reduction devices), which are structurally implemented and documented in the intensive care setting, leaving little room for misclassification of the intervention status.

**Figure 2:**
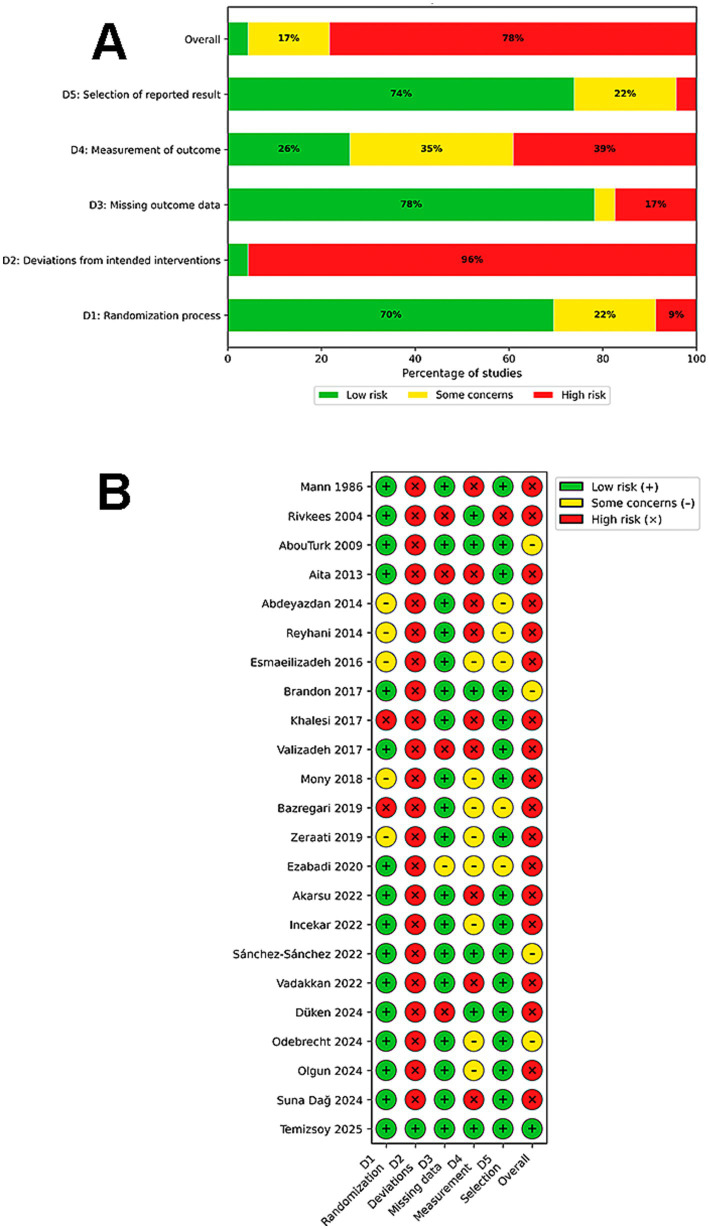
Risk of bias assessment for randomized controlled trials using the Cochrane RoB 2 tool. **(A)** Weighted summary plot of the overall risk of bias across domains (*n* = 23). **(B)** Traffic light plot showing the risk of bias judgment for each individual study.

**Figure 3:**
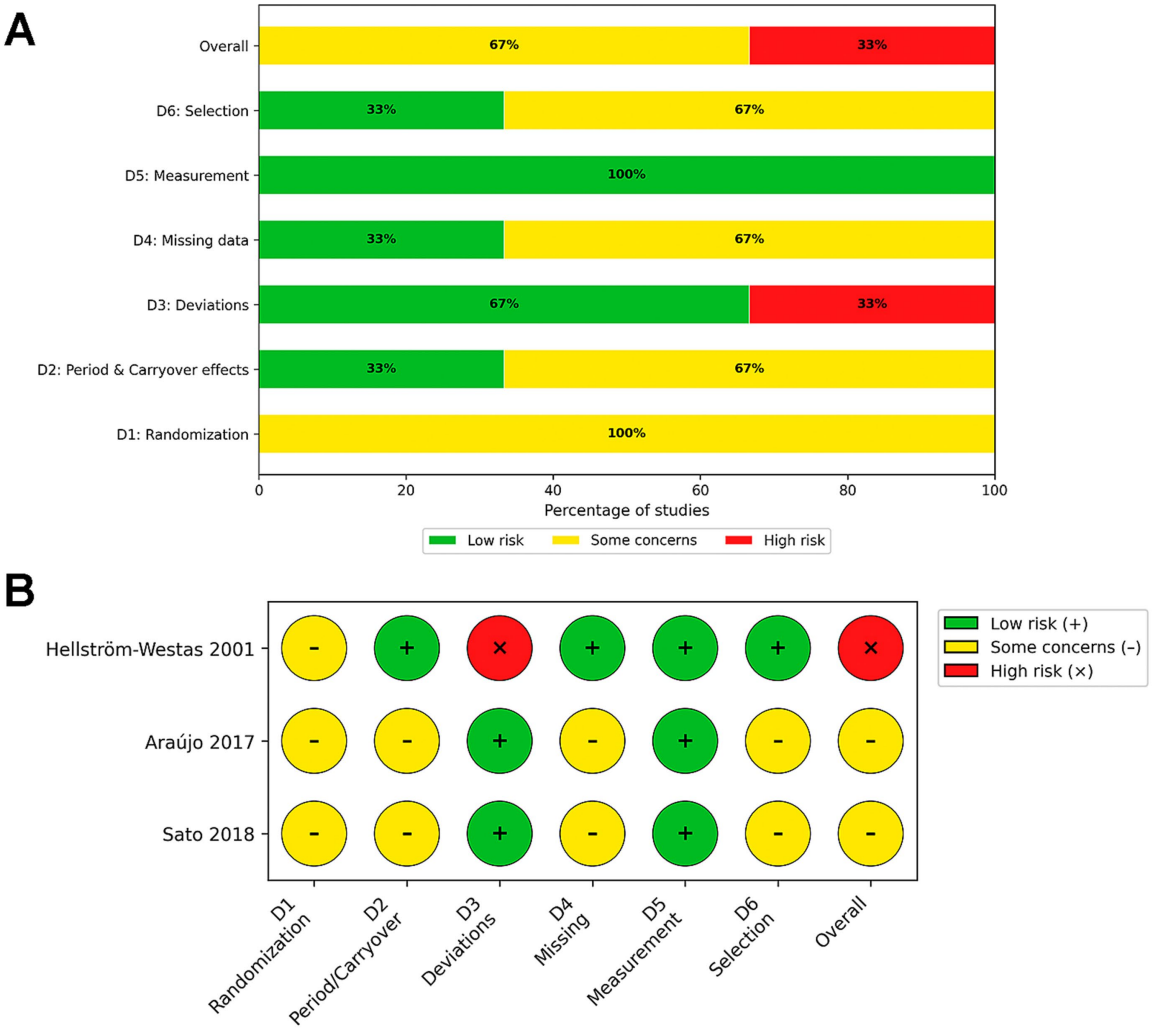
Risk of bias assessment for crossover randomized controlled trials using the Cochrane RoB 2 tool. **(A)** Weighted summary plot illustrating the overall proportion of studies classified as low risk, some concerns, or high risk of bias across each domain. **(B)** Traffic light plot presenting the specific risk of bias judgments for each individual crossover study included in the review.

**Figure 4:**
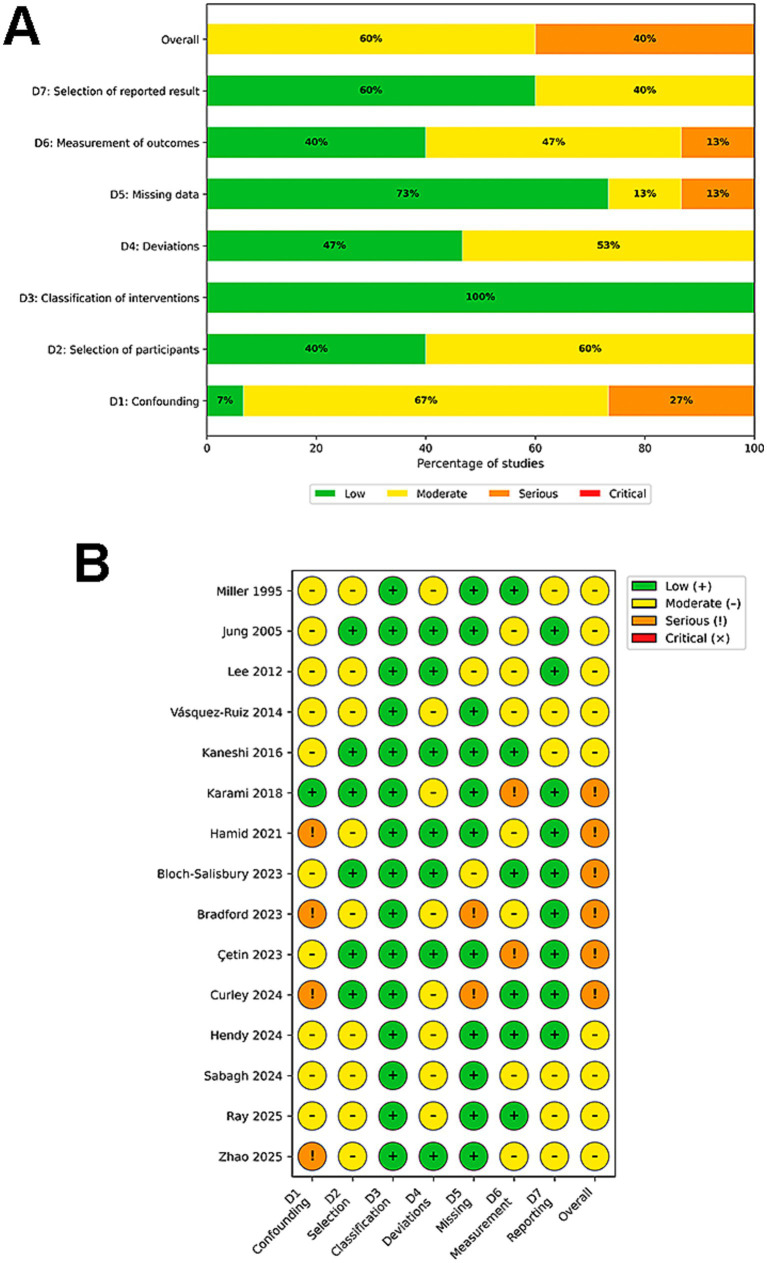
Risk of bias assessment for non-randomized studies using the ROBINS-I tool. **(A)** Weighted summary plot illustrating the overall proportion of studies classified as low, moderate, serious, or critical risk of bias across each domain. **(B)** Traffic light plot presenting the specific risk of bias judgments for each individual non-randomized study included in the review.

The results of the risk of bias judgments for each study and domain are presented in Figures 2–4.

### 3.4 Results of syntheses

A total of 41 studies involving 2,548 participants met the inclusion criteria. Due to variations in data reporting and outcome metrics, the synthesis was conducted in two parts: a quantitative meta-analysis of 16 studies (41, 43, 46, 51, 52, 54, 56, 57, 59–61, 63–65, 67, 80) that provided comparable mean and standard deviation data, and a narrative synthesis (SWiM) (38) of 16 studies that utilized non-parametric or incompatible metrics (47–50, 53, 55, 69–75, 77–79). The remaining 9 studies (42, 44, 45, 58, 62, 66, 68, 76, 81) were excluded from the outcome synthesis due to insufficient statistical reporting (e.g., missing variance) but contributed to the qualitative description of study characteristics. The distribution of studies across these synthesis groups is detailed in Supplementary Material 2.

#### 3.4.1 Effect of circadian interventions on physiological parameters

**Figure 5:**
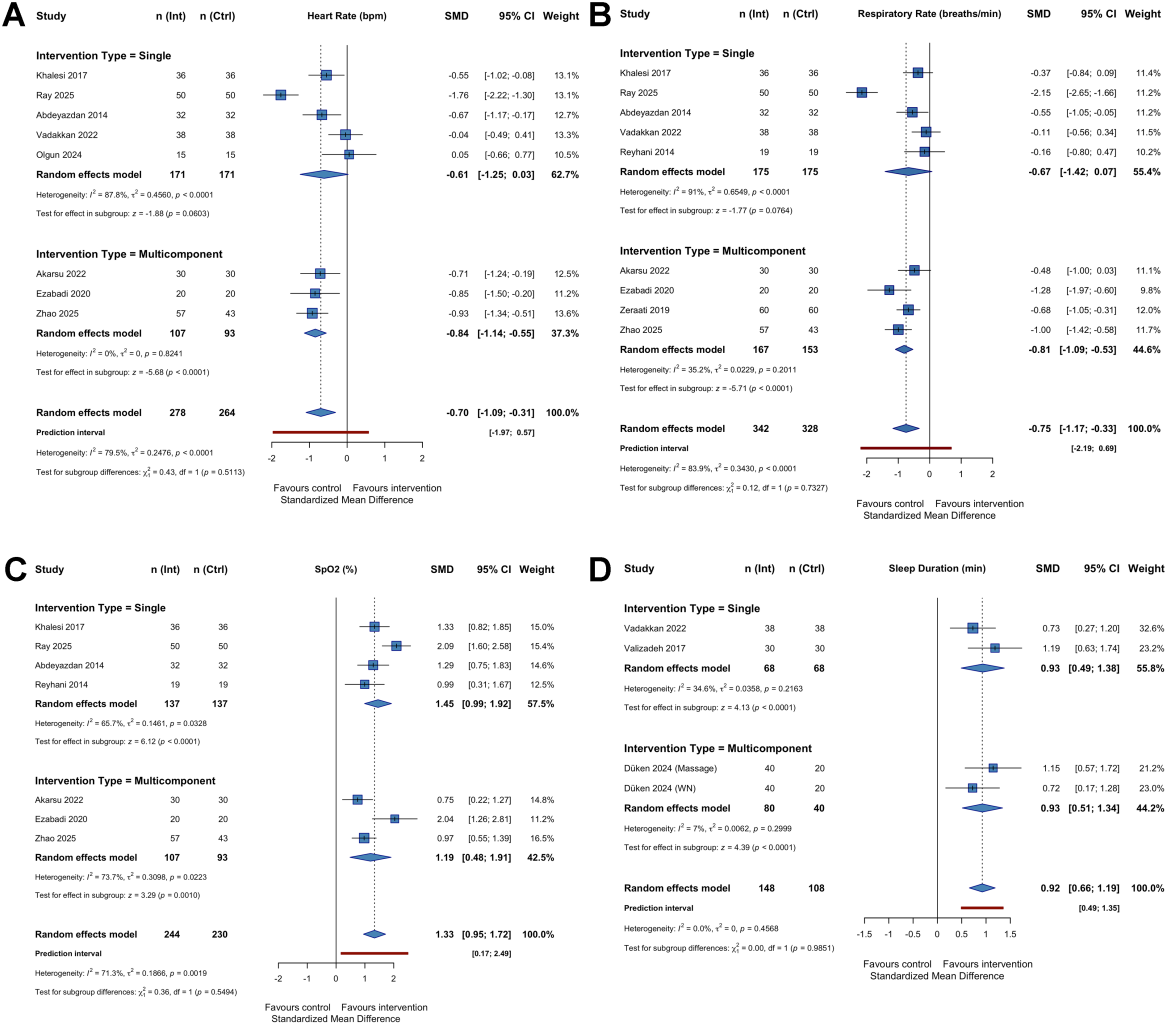
Forest plots illustrating the effects of circadian-tailored interventions versus standard care on physiological stability and sleep outcomes in hospitalized infants. Panels show the pooled meta-analysis results for **(A)** heart rate, **(B)** respiratory rate, **(C)** oxygen saturation (SpO_2_), and **(D)** total sleep duration. In each plot, individual study effect sizes are shown as squares with horizontal lines representing 95% confidence intervals (CIs). The diamond at the bottom represents the pooled standardized mean difference (SMD) and its 95% CI calculated using a random-effects model. For heart rate and respiratory rate (A, B), values to the left of the zero line indicate a reduction favoring the circadian intervention. Conversely, for SpO_2_ and sleep duration **(C,D)**, values to the right of the zero line indicate an increase favoring the intervention. The *I^2^* statistic denotes the degree of statistical heterogeneity across studies. For multi-arm trials sharing a control group, the control sample was split to avoid unit-of-analysis errors, as recommended by the Cochrane Handbook.

The effect of circadian-tailored interventions on physiological parameters was evaluated in 11 studies. For heart rate, the pooled analysis of 8 studies (*n* = 542) (41, 43, 46, 54, 57, 59, 60, 63) demonstrated a significant reduction in the intervention group compared to standard care (SMD -0.70; 95% CI [−1.10, −0.30]; *p* < 0.001). As shown in Figure 5A, significant heterogeneity was observed among the studies (*I^2^* = 79.6%). Similarly, the analysis of respiratory rate in 9 studies (*n* = 670) (41, 43, 46, 57, 59–61, 63, 67) revealed a significant decrease in infants receiving circadian care (SMD = −0.75; 95% CI: [−1.16 to −0.35]; *p* < 0.001), with high heterogeneity (*I^2^* = 84.1%) (Figure 5B). Regarding oxygenation, the pooled results from 7 studies (*n* = 474) (41, 43, 57, 59–61, 63) indicated that oxygen saturation (SpO2) was significantly higher in the intervention group (SMD = 1.33; 95% CI: [0.95 to 1.72]; *p* < 0.001). Heterogeneity for this outcome was also substantial (*I^2^* = 71.7%), as illustrated in Figure 5C. Sensitivity analyses were performed by sequentially removing individual studies, but the statistical significance of the pooled effect sizes remained robust across all three physiological outcomes. A summary of the analysis can be found in Supplementary Material 3.

#### 3.4.2 Effect of circadian interventions on sleep duration

The impact of circadian interventions on total sleep duration was assessed in 3 studies involving 256 participants. The meta-analysis showed that infants in the intervention group had significantly longer sleep duration compared to those in the control group, with a large effect size (SMD = 0.92; 95% CI:[0.66–1.19]; *p* < 0.0001). In contrast with the heterogeneity for the physiological outcomes, sleep duration exhibited no heterogeneity (*I^2^* = 0%) as described in Figure 5D, which shows consistency among the studies.

#### 3.4.3 Subgroup analysis: single vs. multicomponent interventions

To investigate potential sources of heterogeneity and compare intervention strategies, a subgroup analysis was conducted. We found no statistically significant difference between single-component interventions and multicomponent bundles for any of the measured outcomes (*p* > 0.05) (see Table 2). However, it is crucial to note that this review was not designed or powered to formally demonstrate non-inferiority or equivalence. The absence of a statistically significant difference may reflect limited statistical power, high clinical heterogeneity, and the low certainty of the current evidence, rather than true formal equivalence.

**Table 2:** Subgroup analysis comparing the effects of single-component versus multicomponent circadian interventions.

Outcome	Single (*K*)	Single SMD [95% CI]	Multi (*K*)	Multi SMD [95% CI]	p-subgroup
Heart rate	5	−0.61 [−1.26, 0.04]	3	−0.85 [−1.14, −0.55]	0.518
Respiratory rate	5	−0.67 [−1.43, 0.08]	4	−0.82 [−1.11, −0.52]	0.730
SpO2	4	+1.45 [0.98, 1.92]	3	+1.19 [0.55, 1.82]	0.502
Sleep duration	2	+0.93 [0.49, 1.38]	2	+0.95 [0.50, 1.39]	0.965

#### 3.4.4 Narrative synthesis (SWiM)

Sixteen studies (*n* = 786) (47–50, 53, 55, 69–75, 77–79) were synthesized narratively using vote counting based on the direction of effect. Consistent with the meta-analysis results, 75% (12/16) of these studies reported findings favoring the intervention group (47–49, 53, 55, 69, 70, 73, 75, 77–79), while 18.8% (3/16) found no significant difference (71, 72, 74), and 6.2% (1/16) reported partial benefits (50). No study reported results favoring the control condition. Specifically, interventions using cycled lighting (*n* = 3) consistently reported improved weight gain and earlier oral feeding (69, 75, 79). In contrast, results for individual noise reduction (e.g., earmuffs) were mixed, with some studies noting reduced cortisol levels (73) but others finding no effect on weight gain (71). These findings are summarized in in Table 3.

**Table 3:** Summary of findings for studies synthesized narratively using the synthesis without meta-analysis (SWiM) reporting items.

Study	Intervention type	Sample (N)	Key outcome measured	Main finding	Direction
Light cycling and reduction					
Mann et al. (1986) (75)	Cycled Light/Noise	41	Sleep time; Weight gain	Increased sleep duration; 0.5 kg greater weight gain at 3 mo (p < 0.02).	Favors (↑)
Miller et al. (1995) (69)	Cycled Light	41	Weight gain rate	Higher rate of weight gain (14% vs. 7.4%/week; p < 0.05).	Favors (↑)
Rivkees et al. (2004) (70)	Cycled Light	38	Rest-activity patterns	Earlier development of circadian rest-activity distinct patterns.	Favors (↑)
Jung (2005) (79)	Cycled Light	20	Weight; SpO2	Increased weight gain and SpO2; decreased heart rate.	Favors (↑)
Hellström-Westas et al. (2001) (77)	Incubator Cover	18	Sleep states (EEG)	Increased quiet sleep duration during covered periods.	Favors (↑)
Brandon et al. (2017) (72)	Cycled Light (Early)	121	Growth velocity	No significant difference in growth between early vs. late cycling.	No Diff (↔)
Çetin and Ekici (2023) (49)	Incubator Cover	60	SpO2; Vital signs	Higher SpO2 with cover (median 98% vs. 97%; *p* < 0.05).	Favors (↑)
Odebrecht et al. (2024) (53)	Eye Masks (Night)	40	Length of stay	Earlier hospital discharge (median 8 vs. 12 days; *p* = 0.025).	Favors (↑)
Noise reduction					
Abou Turk et al. (2009) (71)	Silicone Earplugs	34	Weight gain; LOS	No significant difference in weight gain or length of stay.	No Diff (↔)
Sato et al. (2018) (74)	Earmuffs	24	Sleep states	No significant difference in total sleep time or sleep efficiency.	No Diff (↔)
Araújo et al. (2017) (73)	Earmuffs	14	Salivary cortisol	Significant reduction in salivary cortisol levels during use.	Favors (↑)
Bloch-Salisbury et al. (2023) (47)	Hearing Protection	10	Sleep percentage	14.1% increase in sleep duration with protection (*p* = 0.02).	Favors (↑)
Multicomponent and Other					
Kaneshi et al. (2016) (78)	Red Light (Night)	48	Sleep/Wake Rhythm	Facilitated development of sleep–wake rhythm (*p* < 0.01).	Favors (↑)
Bradford et al. (2023) (48)	Melatonin	55	Opioid use	Significant reduction in opioid utilization and pain scores (p = 0.049).	Favors (↑)
Curley et al. (2024) (50)	RESTORE Bundle	56	Activity Ratio (DARE)	Improved daytime activity ratio pre-extubation (*p* = 0.04).	Partial (↑)
Sabagh and Ghaljaei (2024) (55)	Quiet Time Protocol	62	Deep Sleep	58% of intervention group achieved deep sleep vs. 0% controls (*p* < 0.001).	Favors (↑)

#### 3.4.5 Reporting bias assessment

As the number of included studies in our meta-analysis was fewer than 10 for all primary outcomes. Specifically, Heart Rate (*k* = 8), Respiratory Rate (*k* = 9), Oxygen Saturation (*k* = 7), and Sleep Duration (*k* = 3), funnel plots and Egger’s regression test were not performed to assess publication bias. According to Cochrane guidelines, tests for funnel plot asymmetry are generally not recommended when there are fewer than 10 studies, as the test power is insufficient to distinguish chance from real asymmetry (35). Consequently, the potential impact of publication bias on the pooled effect sizes could not be formally evaluated.

#### 3.4.6 Certainty assessment

The certainty of the evidence was assessed using the Grading of Recommendations Assessment, Development, and Evaluation (GRADE) approach (40). Overall, the certainty of the body of evidence was judged to be low for all primary outcomes. For the physiological parameters (heart rate, respiratory rate, and oxygen saturation), the evidence was downgraded from high to low primarily due to serious risk of bias. Specifically, performance bias related to the lack of blinding in environmental interventions and serious inconsistency, as indicated by the substantial statistical heterogeneity (*I^2^* > 70%) observed across the included studies. Regarding sleep duration, although inconsistency was not a concern, the evidence was similarly graded as low due to serious risk of bias and serious imprecision, attributable to the limited number of studies (*k* = 4) and the relatively small pooled sample size (*N* = 296). Despite the low certainty ratings, the direction of effect was consistent across all outcomes, with statistically significant benefits observed in the intervention groups. A detailed summary of the GRADE assessment, including the rationale for downgrading each outcome, is presented in Table 4.

**Table 4:** GRADE summary of findings for the comparison of circadian-tailored interventions versus standard care on physiological stability and sleep outcomes in hospitalized infants.

Outcome	Studies	Participants	Effect (SMD)	95% CI	Certainty	Level	Interpretation
Heart Rate	8	542	−0,7	[−1.10, −0.30]	⊕⊕◯◯	LOW^a,b^	Circadian interventions may reduce heart rate in preterm infants (low certainty evidence)
Respiratory Rate	9	670	−0,75	[−1.16, −0.35]	⊕⊕◯◯	LOW^a,b^	Circadian interventions may reduce respiratory rate in preterm infants (low certainty evidence)
Oxygen Saturation (SpO2)	7	474	1,33	[0.95, 1.72]	⊕⊕◯◯	LOW^a,b^	Circadian interventions may improve oxygen saturation in preterm infants (low certainty evidence)
Sleep Duration	4	256	0,92	[0.66, 1.19]	⊕⊕◯◯	LOW^a,c^	Circadian interventions may increase sleep duration in preterm infants (low certainty evidence)

Certainty: ⊕⊕⊕⊕ HIGH, ⊕⊕⊕◯, MODERATE ⊕⊕◯◯, LOW ⊕◯◯◯ VERY LOW.

CI, Confidence Interval; SMD, Standardized Mean Difference; GRADE, Grading of Recommendations Assessment, Development and Evaluation.

GRADE Working Group grades of evidence: High certainty: We are very confident that the true effect lies close to that of the estimate of the effect. Low certainty: Our confidence in the effect estimate is limited; the true effect may be substantially different from the estimate of the effect. ^a^Risk of Bias: Downgraded one level because the majority of included studies had high risk of bias, particularly regarding blinding of participants and personnel. ^b^Inconsistency: Downgraded one level due to substantial unexplained statistical heterogeneity (I2 > 70%) observed across studies. ^c^Imprecision: Downgraded one level due to the limited number of studies (*k* = 4) and relatively small total sample size (*N* < 400).

## 4 Discussion

### 4.1 Summary of main findings

This systematic review and meta-analysis represents the first attempt to directly compare single-component versus multicomponent circadian interventions in neonatal and pediatric intensive care units. Analyzing 41 studies spanning nearly four decades (1986–2025), our meta-analysis demonstrated that circadian interventions significantly modulated all four primary physiological outcomes: reducing heart rate and respiratory rate, while increasing oxygen saturation, and sleep duration (*p* < 0.001). Importantly, while our GRADE assessment rated the certainty of evidence as “low” primarily due to the inherent difficulty of blinding environmental interventions (36) and heterogeneity, this rating indicates limited confidence in the precise magnitude of effects, not in the effectiveness of the interventions themselves (39).

### 4.2 Single vs. combined interventions in neonates: an ontogenic perspective

The central finding of this review is the absence of significant differences between single-component and multicomponent interventions across all outcomes (*p* > 0.05). This challenges the intuitive assumption that comprehensive “bundles” yield superior results compared to focused approaches. A critical consideration in interpreting this finding is the developmental stage of the circadian system. The suprachiasmatic nucleus (SCN) is present by 18 weeks of gestation but undergoes substantial postnatal maturation (83, 84). Critically, the neonatal SCN contains only ~13% of the adult number of vasopressin-expressing neurons, and adult levels are not attained until 2–3 years of life (6, 85). This developmental immaturity has profound implications. During the sensitive window of prematurity, the plastic circadian system may respond more robustly to a single, strong zeitgeber (like light) than to multiple, competing signals (7, 86). This “less is more” hypothesis is supported by Van Gilst et al. (13), who demonstrated that preterm infants exposed to cycled light showed earlier emergence of sleep–wake rhythms compared to term infants, suggesting a unique sensitivity to photic entrainment. Furthermore, Govindan et al. (81) showed that circadian rhythm amplitude increases as a function of postnatal age, emphasizing the role of ex-utero maturation. Therefore, in an immature system, providing a clear, consistent light–dark cycle may be more effective than introducing multiple zeitgebers simultaneously, which could potentially overwhelm an underdeveloped entrainment pathway. The attractive “less is more” hypothesis (that simple interventions may yield similar benefits to complex ones) appears plausible for the immature neonatal circadian system. However, this finding must be strictly restricted to neonatal populations. It should not be extrapolated to older pediatric patients (PICU) or adults, whose mature circadian systems likely require more comprehensive, multicomponent approaches to overcome ICU disruption.

### 4.3 Complexity does not guarantee effectiveness: the case for cycled light

Consistent with the developmental hypothesis, cycled light emerged as a particularly robust intervention. Despite heterogeneity in implementation including variations in light intensity (ranging from <30 lux to 300–580 lux during daytime), duration of exposure, timing of introduction, and follow-up periods, studies consistently demonstrated favorable outcomes (45, 49, 53, 54, 61, 64, 69, 70, 72, 75, 77, 79–81). The 2024 Cochrane review by Morag et al. (87), while noting the need for larger studies, cautiously supports cycled light implementation in NICUs. The biological rationale for cycled light’s effectiveness is well-established. Light is the primary zeitgeber for the SCN, acting through melanopsin-containing intrinsically photosensitive retinal ganglion cells (ipRGCs) that project directly to the circadian pacemaker (11, 22). Hazelhoff et al. (8) emphasized that while preterm infants may not yet have fully developed photic entrainment pathways, the basic structures of the eye and central clock are present by 24 weeks of gestation. Environmental light input can therefore be conveyed to an SCN that is – at least partly – functional, supporting circadian system maturation during the critical period of NICU hospitalization. This consistency across diverse protocols suggests that the fundamental principle of establishing light–dark cycles may be more important than specific parameters in neonatal populations. For mature circadian systems, however, the quality and spectral composition of light may be more critical. Wasden et al. (88) demonstrated that circadian rhythms are best entrained by light wavelengths between 446 and 477 nanometers – a specificity that may be less relevant to immature systems but crucial for adult circadian alignment.

### 4.4 Challenges in standardization and clinical implications

A significant challenge encountered in this review was the lack of standardized definitions. We identified circadian interventions ranging from simple earmuff application to comprehensive chronotherapeutic bundles. Some included interventions may not be purely circadian in nature; noise reduction with earmuffs primarily serves as a stress-reduction intervention with secondary circadian benefits (73, 89), and nesting/swaddling have multiple physiological effects beyond circadian modulation (56). At present, many authors are acknowledging this challenge and called for standardized definitions and outcomes measures (27, 28). The minimal reporting guidelines developed by Spitschan et al. (90) for lighting interventions provide a useful starting point. Until such consensus is achieved, systematic reviews in this field will continue to face difficulties in comparing results across studies and populations. Despite these definition challenges, the clinical implications for nursing are clear. Circadian interventions are inherently nursing-driven, positioning nurses as primary implementers (91, 92). The finding that simpler interventions are as effective as complex bundles in neonatal populations has direct implications for nursing workload. Altimier and Phillips (93) developed the Neonatal Integrative Developmental Care Model with seven neuroprotective core measures. Our findings suggest that prioritizing light–dark cycles may yield substantial benefits for neonates without requiring simultaneous implementation of all components. However, this recommendation is age-specific; adult and pediatric populations may require multicomponent approaches.

### 4.5 Strengths and limitations

This review has several strengths. We followed PRISMA 2020 guidelines and registered our protocol prospectively. Our comprehensive search across multiple databases identified 41 studies spanning nearly four decades. The use of both meta-analysis and SWiM narrative synthesis allowed inclusion of studies with diverse reporting formats. Risk of bias was assessed using validated tools (RoB 2 and ROBINS-I), and certainty of evidence was evaluated using GRADE methodology.

However, several limitations must be acknowledged. The substantial heterogeneity observed (*I^2^* = 72–84% for physiological outcomes) limits confidence in pooled estimates and likely reflects true variation in intervention protocols, populations, and outcome measurement timing. Nine studies were excluded from synthesis due to incompatible outcome definitions or insufficient statistical reporting, potentially introducing selection bias. The predominance of studies from middle-income countries may limit generalizability to other healthcare contexts. Publication bias could not be formally assessed due to the small number of studies per outcome, though funnel plot inspection suggested possible asymmetry.

### 4.6 Future directions

Based on our findings, we propose several priorities for future research. First, standardized definitions and reporting guidelines for circadian interventions are urgently needed. The minimal reporting guidelines developed by Spitschan et al. (90) for lighting interventions provide a useful starting point that should be adopted and extended to other circadian domains. Second, adequately powered randomized trials specifically addressing cycled light implementation are warranted. Given the consistent positive findings despite protocol heterogeneity, pragmatic trials testing real-world implementation strategies may be more valuable than efficacy trials with strict protocols. Third, research in pediatric intensive care populations is critically needed. The near-absence of PICU studies represents a significant gap that cannot be addressed by extrapolating neonatal findings. Fourth, studies should incorporate objective circadian biomarkers such as melatonin rhythms, cortisol profiles, or actigraphy to verify that interventions actually achieve circadian alignment rather than relying solely on clinical outcomes. Finally, long-term neurodevelopmental follow-up should be prioritized. While immediate physiological changes are encouraging, the ultimate goal of circadian interventions is to optimize long-term outcomes for this vulnerable population (16).

## 5 Conclusion

This systematic review and meta-analysis demonstrates that circadian-tailored interventions effectively modulate physiological parameters and improve sleep duration in critically ill neonates. A key finding is that single-component interventions, such as cycled lighting alone, showed no statistically significant difference in outcomes compared to complex multicomponent bundles in the NICU setting. This supports a potential ‘less is more’ approach specifically for the developing neonatal circadian system, suggesting that solitary, robust environmental cues may be sufficient to promote entrainment. Clinicians in neonatal units should prioritize the feasible implementation of standardized light–dark cycles and sensory protection. However, due to the critical scarcity of evidence from older pediatric populations, these findings cannot be extrapolated to the PICU. Future research must strictly stratify by age, prioritize primary studies in pediatric intensive care, and focus on standardizing environmental reporting.

## Data Availability

The original contributions presented in the study are included in the article/Supplementary material, further inquiries can be directed to the corresponding author.

## Footnotes

1
https://doi.org/10.17605/OSF.IO/8SPZM

## References

[1] Goel P Goel A. Exploring the evolution of sleep patterns from infancy to adolescence. Cureus. (2024) 16:e64759. doi: 10.7759/cureus.64759, 39156264 PMC11329291

[2] Agostini A Centofanti S. Normal sleep in children and adolescence. Psychiatr Clin North Am. (2024) 47:1–14. doi: 10.1016/j.psc.2023.06.001, 38302199

[3] Li W Ma L Yang G Gan WB. REM sleep selectively prunes and maintains new synapses in development and learning. Nat Neurosci. (2017) 20:427–37. doi: 10.1038/nn.4479, 28092659 PMC5535798

[4] de Vivo L Bellesi M. The role of sleep and wakefulness in myelin plasticity. Glia. (2019) 67:2142–52. doi: 10.1002/glia.23667, 31237382 PMC6771952

[5] Luu P Tucker DM. Continuity and change in neural plasticity through embryonic morphogenesis, fetal activity-dependent synaptogenesis, and infant memory consolidation. Dev Psychobiol. (2023) 65:e22439. doi: 10.1002/dev.22439, 38010309

[6] Wong SD Wright KP Spencer RL Vetter C Hicks LM Jenni OG . Development of the circadian system in early life: maternal and environmental factors. J Physiol Anthropol. (2022) 41:22. doi: 10.1186/s40101-022-00294-0, 35578354 PMC9109407

[7] Rivkees SA. Developing circadian rhythmicity in infants. Pediatrics. (2003) 112:373–9. doi: 10.1542/peds.112.2.373 12897290

[8] Hazelhoff EM Dudink J Meijer JH Kervezee L. Beginning to see the light: lessons learned from the development of the circadian system for optimizing light conditions in the neonatal intensive care unit. Front Neurosci. (2021) 15:634034. doi: 10.3389/fnins.2021.634034, 33815040 PMC8013699

[9] Byeon AGH Weiss SK Gilfoyle E McKinnon NK. Sleep fragmentation in critically ill children: a review of contributing factors in the pediatric intensive care unit and neurodevelopmental outcomes. Front Sleep. (2025) 4:1–13. doi: 10.3389/frsle.2025.1629408, 41425199 PMC12713949

[10] Hassinger AB Afzal S Rauth M Breuer RK. Pediatric intensive care unit related sleep and circadian dysregulation: a focused review. Semin Pediatr Neurol. (2023) 48:101077. doi: 10.1016/j.spen.2023.101077, 38065630

[11] Allada R Bass J. Circadian mechanisms in medicine. N Engl J Med. (2021) 384:550–61. doi: 10.1056/nejmra1802337, 33567194 PMC8108270

[12] Roenneberg T Merrow M. The circadian clock and human health. Curr Biol. (2016) 26:R432–43. doi: 10.1016/j.cub.2016.04.011, 27218855

[13] Van Gilst D Puchkina AV Roelants JA Kervezee L Dudink J Reiss IKM . Effects of the neonatal intensive care environment on circadian health and development of preterm infants. Front Physiol. (2023) 14:1. doi: 10.3389/fphys.2023.1243162, 37719464 PMC10500197

[14] Dijk DJ Duffy JF Silva EJ Shanahan TL Boivin DB Czeisler CA. Amplitude reduction and phase shifts of melatonin, cortisol and other circadian rhythms after a gradual advance of sleep and light exposure in humans. PLoS One. (2012) 7:e30037. doi: 10.1371/journal.pone.0030037, 22363414 PMC3281823

[15] Bano-Otalora B Martial F Bechtold DA Allen AE Brown TM Belle D MDC LRJ. Bright daytime light enhances circadian amplitude in a diurnal mammal. Proc Natl Acad Sci USA. (2021) 118:e2100094118. doi: 10.1073/pnas.2100094118/-/DCSupplemental 34031246 PMC8179182

[16] Van der Linden IA Hazelhoff EM De Groot ER Vijlbrief DC Schlangen LJM De Kort YAW . Characterizing light-dark cycles in the neonatal intensive care unit: a retrospective observational study. Front Physiol. (2023) 14:1217660. doi: 10.3389/fphys.2023.1217660, 37664437 PMC10469299

[17] Greenfield KD Karam O Iqbal O’Meara AM. Brighter days may be ahead: continuous measurement of pediatric intensive care unit light and sound. Front Pediatr. (2020) 8:590715. doi: 10.3389/fped.2020.590715 33194924 PMC7649178

[18] Ruben MD Francey LJ Guo Y Wu G Cooper EB Shah AS . A large-scale study reveals 24-h operational rhythms in hospital treatment. Proc Natl Acad Sci USA. (2019) 116:20953–8. doi: 10.1073/pnas.1909557116, 31575744 PMC6800314

[19] Vetter C. Circadian disruption: what do we actually mean? Eur J Neurosci. (2018) 51:531–50. doi: 10.1111/ejn.14255, 30402904 PMC6504624

[20] Fishbein AB Knutson KL Zee PC. Circadian disruption and human health. J Clin Invest. (2021) 131:e148286. doi: 10.1172/JCI148286, 34596053 PMC8483747

[21] Berglund B Lindvall T Schwela DH, World Health Organization, Occupational and Environmental Health Team. Guidelines for Community Noise. Geneva, Switzerland: World Health Organization (WHO). (1999).

[22] Panda S. The arrival of circadian medicine. Nat Rev Endocrinol. (2019) 15:67–9. doi: 10.1038/s41574-018-0142-x, 30602736

[23] Kramer A Lange T Spies C Finger AM Berg D Oster H. Foundations of circadian medicine. PLoS Biol. (2022) 20:e3001567. doi: 10.1371/journal.pbio.3001567, 35324893 PMC8946668

[24] Luther R McLeod A. The effect of chronotherapy on delirium in critical care – a systematic review. Nurs Crit Care. (2018) 23:283–90. doi: 10.1111/nicc.12300, 28508438

[25] Luetz A Spies C Kervezee L. It’s about time: circadian medicine in the intensive care unit. Intensive Care Med. (2024) 50:283–6. doi: 10.1007/s00134-023-07297-0, 38112772

[26] Hatfield LA Murphy N Karp K Polomano RC. A systematic review of behavioral and environmental interventions for procedural pain management in preterm infants. J Pediatr Nurs. (2019) 44:22–30. doi: 10.1016/j.pedn.2018.10.004, 30683278

[27] Hiemstra FW González LB Engelhardt LJ Hancke L Pilz LK Adler AI . Challenges and recommendations for integrating circadian medicine in critical care: a roadmap. Chest. (2025). doi: 10.1016/j.chest.2025.12.010 PMC13083041 41429288

[28] Knauert MP Ayas NT Anderson BJ Bosma KJ Cordoza ML Devlin JW . Causes, consequences, and treatments of sleep and circadian disruption in the ICU: an official American Thoracic Society research statement. Am J Respir Crit Care Med American Thoracic Society. (2023) 207:E49–68. doi: 10.1164/rccm.202301-0184ST, 36999950 PMC10111990

[29] Lewis P Wild U Pillow JJ Foster RG Erren TC. A systematic review of chronobiology for neonatal care units: what we know and what we should consider. Sleep Med Rev. (2024) 73:101872. doi: 10.1016/j.smrv.2023.101872, 38000120

[30] Li J Fan Y Luo R Yin N Wang Y Jing J . The impact of non-pharmacological sleep interventions on delirium prevention and sleep improvement in postoperative ICU patients: a systematic review and network Meta-analysis. Intensive Crit Care Nurs. (2025) 87:103925. doi: 10.1016/j.iccn.2024.103925, 39709722

[31] Zhang S Han Y Xiao Q Li H Wu Y. Effectiveness of bundle interventions on ICU delirium: a meta-analysis. Crit Care Med. (2021) 49:335–46. doi: 10.1097/CCM.0000000000004773, 33332818 PMC7803454

[32] Page MJ McKenzie JE Bossuyt PM Boutron I Hoffmann TC Mulrow CD . The PRISMA 2020 statement: an updated guideline for reporting systematic reviews. BMJ. (2021) 372:n71. doi: 10.1136/bmj.n71, 33782057 PMC8005924

[33] Corrotea-Maltez CC Fernández-Flores CV Gutierrez-Neira CL Nirrian-Pérez AJ Mansilla-Muñoz J Bustos-González L Effectiveness of combined versus single circadian interventions in pediatric intensive care units: a systematic review protocol protocol for a systematic review of circadian interventions in PICUs [Epubh ahead of preprint]. (2025) doi: 10.1101/2025.08.19.25334031 PMC13056654 41959633

[34] O’Connor D Green S Higgins JP. "Defining the review question and developing criteria for including studies". In: Higgins JP Green S, editors. Cochrane Handbook for Systematic Reviews of Interventions. Chichester, UK: John Wiley & Sons, Ltd. (2008). p. 81–94.

[35] Higgins JP Thomas J Chandler J Cumpston M Li T Page MJ . Cochrane Handbook for Systematic Reviews of Interventions. 2nd ed. Chichester (UK): John Wiley & Sons (2019). p. 1–659.

[36] Sterne JAC Savović J Page MJ Elbers RG Blencowe NS Boutron I . RoB 2: a revised tool for assessing risk of bias in randomised trials. BMJ. (2019) 366:l4898. doi: 10.1136/bmj.l4898 31462531

[37] Sterne JA Hernán MA Reeves BC Savović J Berkman ND Viswanathan M . ROBINS-I: a tool for assessing risk of bias in non-randomised studies of interventions. BMJ. (2016) 355:i4919. doi: 10.1136/bmj.i4919 27733354 PMC5062054

[38] Campbell M McKenzie JE Sowden A Katikireddi SV Brennan SE Ellis S . Synthesis without meta-analysis (SWiM) in systematic reviews: reporting guideline. BMJ. (2020) 367:l6890. doi: 10.1136/bmj.l6890 PMC7190266 31948937

[39] Guyatt GH Oxman AD Schünemann HJ Tugwell P Knottnerus A. GRADE guidelines: a new series of articles in the journal of clinical epidemiology. J Clin Epidemiol. (2011) 64:380–2. doi: 10.1016/j.jclinepi.2010.09.011, 21185693

[40] Schünemann H Brożek J Guyatt G Oxman A. (2013). GRADE Handbook for Grading Quality of Evidence and Strength of Recommendations. Available online at: https://gdt.guidelinedevelopment.org/app/handbook/handbook.html (accessed January 28, 2026)

[41] Ezabadi AR Dehghani K Fallahzade H. Effect of the artificial night with facilitated tucking and artificial night alone on the physiological indices of premature infants. Iran J Neonatol. (2020) 11:91–8. doi: 10.22038/ijn.2020.42009.1696

[42] Abdel Hamid TA Abdel Latif DK Bakeer A Ibrahim AA Nasef KA. Effect of light and noise on physiological parameters in a sample of preterm neonates in the neonatal intensive care of Cairo University teaching hospital. Iran J Neonatol. (2021) 12:37–45. doi: 10.22038/ijn.2021.51741.1941

[43] Akarsu Ö Balci S. The effect of a noise and light-reducing hat on the comfort and physiologic parameters of the preterm neonates. Ann Clin Anal Med. (2022) 13:1092–7. doi: 10.4328/acam.21219

[44] Íncekar, MC, Gözen D Tastekin A. The effect of light and noise reduction on the sleep state of preterm infants. Ann Clin Anal Med (2022) 13:914–918. doi:doi: 10.4328/acam.21162

[45] Sánchez-Sánchez M García TL Heredia D Reséndiz I Cruz L Santiago J . Effect of a light-darkness cycle on the body weight gain of preterm infants admitted to the neonatal intensive care unit. Sci Rep. (2022) 12:17833. doi: 10.1038/s41598-022-22533-1, 36266474 PMC9584226

[46] Vadakkan AJ Prabakaran V. Comparison of the effect of nesting and swaddling on sleep duration and arousal frequency among preterm neonates: a randomized clinical trial. J Caring Sci. (2022) 11:126–31. doi: 10.34172/jcs.2022.17, 36247038 PMC9526794

[47] Bloch-Salisbury E McKenna L Boland E Chin D. Assessment of a hearing protection device on infant sleep in the neonatal intensive care unit. J Sleep Res. (2023) 32:e13610. doi: 10.1111/jsr.13610, 35460141 PMC9589402

[48] Bradford C Miller JL Harkin M Chaaban H Neely SB Johnson PN. Melatonin use in infants admitted to intensive care units. J Pediatr Pharmacol Ther. (2023) 28:635–42. doi: 10.5863/1551-6776-28.7.635, 38025149 PMC10681084

[49] Çetin K Ekici B. The effect of incubator cover on newborn vital signs: the Design of Repeated Measurements in two separate groups with no control group. Children. (2023) 10:1224. doi: 10.3390/children10071224, 37508721 PMC10378478

[50] Curley MAQ Dawkins-Henry OS Kalvas LB Perry-Eaddy MA Georgostathi G Yuan I . The nurse-implemented Chronotherapeutic bundle in critically ill children, RESTORE resilience (R2): pilot testing in a two-phase cohort study, 2017–2021. Pediatr Crit Care Med. (2024) 25:1051–64. doi: 10.1097/PCC.0000000000003595, 39133067 PMC11534519

[51] Düken ME Yayan EH. The effects of massage therapy and white noise application on premature infants’ sleep. Explore. (2024) 20:319–27. doi: 10.1016/j.explore.2023.09.002, 37806925

[52] Hendy A Alsharkaw SS El-Nagger NS Hendy A Sayed S Nashwan AJ. Outcome of creating clustering nursing care and healing environment on premature infants’ behavioural outcomes. BMJ Paediatr Open. (2024) 8:e002716. doi: 10.1136/bmjpo-2024-002716, 38977353 PMC11340664

[53] Vergne O de Abreu AC Braga A de Oliveira M Alquati T Tonon AC . Use of light protection equipment at night reduces time until discharge from the neonatal intensive care unit: a randomized interventional study. J Biol Rhythm. (2024) 39:68–78. doi: 10.1177/07487304231201752, 37846856

[54] Olgun AB Yüksel D Yardımcı F. The effect of a light-dark cycle on premature infants in the neonatal intensive care unit: a randomized controlled study. J Pediatr Nurs. (2024) 77:e343–9. doi: 10.1016/j.pedn.2024.04.050 38724313

[55] Sabagh K Ghaljaei F. The interventional effect of quiet time protocol on the sleep status of premature neonates admitted to the NICU. Iran J Neonatol. (2024) 15:53–62. doi: 10.22038/IJN.2023.73016.2414

[56] Suna Dağ Y Yayan EH. The effect of facilitated tucking and white noise on stress and sleep of newborns receiving nasal continuous positive airway pressure. J Pediatr Nurs. (2024) 77:e442–9. doi: 10.1016/j.pedn.2024.05.008, 38729891

[57] Ray SS Wilson LP Khan Z Patil PN. Effect of earmuff use on physiological and behavioral responses in preterm neonates: a non-randomized, controlled, before-after, quality improvement, observational prospective cohort study. Eur J Pediatr. (2025) 184:217. doi: 10.1007/s00431-025-06055-2, 40024939

[58] Temizsoy E Uysal G Karadag N. The effect of a Chronobiological feeding model on growth parameters and length of hospitalization in preterm infants: a randomized controlled study. Breastfeed Med. (2025) 20:432–40. doi: 10.1089/bfm.2024.0221, 40195944

[59] Zhao W Li H Guo H Yuan E Cao F Shi Y . Application of music intervention combined with multidimensional nursing in very low birth weight infants: a retrospective cohort study. Noise Health. (2025) 27:422–9. doi: 10.4103/nah.nah_33_25, 40932077 PMC12459712

[60] Abdeyazdan Z Ghassemi S Marofi M. The effects of earmuff on physiologic and motor responses in premature infants admitted in neonatal intensive care unit. Iran J Nurs Midwifery Res. (2014) 19:107–12. 24834077 PMC4020017

[61] Reyhani T Aemmi SZ Sannadgol V Boskabadi H. The effect of creating an artificial night on physiological changes in preterm infants. Int J Pediatr. (2014) 2:4–7. Available online at: http://ijp.mums.ac.ir/article_3361.html

[62] Esmaeilizadeh M Shoja M Shoja E Shoja M Nejat H Oudi D. Comparing the effects of continuous and cyclical lightings on weight gain and length of hospital stay among preterm neonates. Mod Care J. (2016) 13:12–18. doi: 10.17795/modernc.8951

[63] Khalesi N Khosravi N Ranjbar A Godarzi Z Karimi A. The effectiveness of earmuffs on the physiologic and behavioral stability in preterm infants. Int J Pediatr Otorhinolaryngol. (2017) 98:43–7. doi: 10.1016/j.ijporl.2017.04.028, 28583502

[64] Valizadeh S Hosseini M Jafarabadi MA Mirnia K Saeidi F Jabraeeli M. Comparison of 2 methods of light reduction on preterm infants’ sleep pattern in NICU: a randomized controlled trial. Crescent J Med Biol Sci. (2017) 4:211–6. doi: 10.22038/cjmb.2017.54593

[65] Karami S Marofi M. Comparison between the effects of environmental and behavioral interventions on sleep cycle of preterm infants in NICU. J Kerman Univ Med Sci. (2018) 25:505–8. Available online at: https://jkmu.kmu.ac.ir/article_87042.html

[66] Bazregari M Mirlashari J Ranjbar H Pouraboli B. Effect of clustered nursing care on sleep behaviors of the preterm neonates admitted to the neonatal intensive care unit. Iran J Neonatol. (2019) 10:14–20. doi: 10.22038/ijn.2019.34814.1516

[67] Zeraati H Nasimi F Ghorbanzadeh M Sarani A. Effects of a quiet time protocol implementation on respiratory rate and SpO2 in preterm infants. Shiraz E Medical Journal. (2019) 20:e84063. doi: 10.5812/semj.84063

[68] Mony K Selvam V Diwakar K Raghavan VR. Effect of nesting on sleep pattern among preterm infants admitted in NICU. Biomed Res. (2018) 10:1994–7. doi: 10.4066/biomedicalresearch.29-18-326

[69] Miller CL White R Whitman TL O’callaghan MF Maxwell SE. The effects of cycled versus noncycled lighting on growth and development in preterm infants. Infant Behav Dev. (1995) 18:87–95. doi: 10.1016/0163-6383(95)90010-1

[70] Rivkees SA Mayes L Jacobs H Gross I. Rest-activity patterns of premature infants are regulated by cycled lighting. Pediatrics. (2004) 113:833–9. doi: 10.1542/peds.113.4.833. 15060235

[71] Abou Turk C Williams AL Lasky RE. A randomized clinical trial evaluating silicone earplugs for very low birth weight newborns in intensive care. J Perinatol. (2009) 29:358–63. doi: 10.1038/jp.2008.236, 19194455 PMC2674530

[72] Brandon DH Silva SG Park J Malcolm W Kamhawy H Holditch-Davis D. Timing for the introduction of cycled light for extremely preterm infants: a randomized controlled trial. Res Nurs Health. (2017) 40:294–310. doi: 10.1002/nur.21797, 28431191 PMC5522348

[73] Araújo FM Pedreira M d LG Avelar AFM Pradella-Hallinan ML d C Tsunemi MH Pinheiro EM. Sleep and salivary cortisol in preterm neonates: a clinical, randomized, controlled, crossover study. Rev Bras Enferm. (2018) 71:1358–65. doi: 10.1590/0034-7167-2017-0546 29972535

[74] Sato MH Pedreira M d LG Avelar AFM Tsunemi MH Orsi KCSC Pradella-Hallinan ML d C . Influence of ear protectors on the sleep of preterm newborns: a randomized controlled clinical study. Clin Nurs Res. (2020) 29:260–7. doi: 10.1177/1054773818806171 30338694

[75] Mann NP Haddow R Stokes L Goodley S Rutter N. Effect of night and day on preterm infants in a newborn nursery: randomised trial. Br Med J. (1986) 293:1265–7. doi: 10.1136/bmj.293.6557.1265, 3096460 PMC1342106

[76] Aita M Johnston C Goulet C Oberlander TF Snider L. Intervention minimizing preterm infants’ exposure to NICU light and noise. Clin Nurs Res. (2013) 22:337–58. doi: 10.1177/1054773812469223, 23275433

[77] Hellström-Westas L Inghammar M Isaksson K Rosén I Stjernqvist K. Short-term effects of incubator covers on quiet sleep in stable premature infants. Acta Paediatr. (2001) 90:1004–8. doi: 10.1080/080352501316978075, 11683187

[78] Kaneshi Y Ohta H Morioka K Hayasaka I Uzuki Y Akimoto T . Influence of light exposure at nighttime on sleep development and body growth of preterm infants. Sci Rep. (2016) 6:21680. doi: 10.1038/srep21680, 26877166 PMC4753683

[79] Jung IS. Effects of cycled lighting on body weight, physiological variables and behavioral states in low birth weight infants. J Korean Acad Nurs. (2005) 35:143–53. doi: 10.4040/jkan.2005.35.1.143, 15778566

[80] Lee S-J Han K-J Bang K-S. Effects of cycled lighting on circadian rhythms of premature infants. J Korean Acad Child Health Nurs. (2012) 18:85–94. doi: 10.4094/jkachn.2012.18.2.85

[81] Vásquez-Ruiz S Maya-Barrios JA Torres-Narváez P Vega-Martínez BR Rojas-Granados A Escobar C . A light/dark cycle in the NICU accelerates body weight gain and shortens time to discharge in preterm infants. Early Hum Dev. (2014) 90:535–40. doi: 10.1016/j.earlhumdev.2014.04.015, 24831970

[82] Lee C Smith M Eastman C. A compromise phase position for permanent night shift workers: circadian phase after two night shifts with scheduled sleep and light/dark exposure. Chronobiol Int. (2006) 23:859–75. doi: 10.1080/07420520600827160 16887753

[83] Swaab DF Hofman MA Honnebier MBOM. Development of vasopressin neurons in the human suprachiasmatic nucleus in relation to birth. Dev Brain Res. (1990) 52:289–93. doi: 10.1016/0165-3806(90)90247-v, 2331797

[84] Reppert SM Weaver DR. Coordination of circadian timing in mammals. Nature. (2002) 418:935–41. doi: 10.1038/nature00965, 12198538

[85] Seron-Ferre M Valenzuela GJ Torres-Farfan C. Circadian clocks during embryonic and fetal development. Birth Defects Res C Embryo Today. (2007) 81:204–14. doi: 10.1002/bdrc.20101, 17963275

[86] Escobar C Rojas-Granados A Angeles-Castellanos M. "Development of the circadian system and relevance of periodic signals for neonatal development". In: Handbook of Clinical Neurology. Amsterdam, Netherlands: Elsevier. (2021). p. 249–58. 10.1016/B978-0-12-819975-6.00015-7 34225966

[87] Morag I Ohlsson A. Cycled light in the intensive care unit for preterm and low birth weight infants (review). Cochrane Database Syst Rev. (2016) 2016:CD006982. doi: 10.1002/14651858.CD006982.pub4 27508358 PMC6464252

[88] Wasden K Intihar T Knauert M. Light spectra: an important consideration for circadian alignment in the MEDICAL ICU. Chest. (2021) 160:A2425. doi: 10.1016/j.chest.2021.07.2096

[89] Sibrecht G Wróblewska-Seniuk K Bruschettini M. Noise or sound management in the neonatal intensive care unit for preterm or very low birth weight infants. Cochrane Database Syst Rev. (2024) 2024:CD010333. doi: 10.1002/14651858.CD010333.pub4 PMC11137833 38813836

[90] Spitschan M Stefani O Blattner P Gronfier C Lockley SW Lucas RJ. How to report light exposure in human chronobiology and sleep research experiments. Clocks Sleep. (2019) 1:280–9. doi: 10.3390/clockssleep1030024, 31281903 PMC6609447

[91] Phillips R Solomon J Dixon L Altimier L. Neuroprotective infant and family-centered developmental Care for the Tiniest Babies. Crit Care Nurs Clin North Am. (2024) 36:167–84. doi: 10.1016/j.cnc.2023.11.003, 38705686

[92] Firmino C Rodrigues M Franco S Ferreira J Simões AR Castro C . Nursing interventions that promote sleep in preterm newborns in the neonatal intensive care units: an integrative review. Int J Environ Res Public Health. (2022) 19:10953. doi: 10.3390/ijerph191710953, 36078666 PMC9518210

[93] Altimier L Phillips R. The neonatal integrative developmental care model: advanced clinical applications of the seven core measures for neuroprotective family-centered developmental care. Newborn Infant Nurs Rev. (2016) 16:230–44. doi: 10.1053/j.nainr.2016.09.030

