# Supplementary material for: Effectiveness of combined versus single circadian interventions in neonatal and pediatric intensive care units: a systematic review with meta-analysis
Source: Front Neurol. 2026 Mar 25;17:1801194. doi: 10.3389/fneur.2026.1801194 (PMC13056654; doi:10.3389/fneur.2026.1801194)
Supplement: Supplementary file 1 [file Data_sheet_1.pdf]

Supplementary Material

Effectiveness of Combined versus Single Circadian Interventions in Neonatal and Pediatric Intensive Care Units: a Systematic Review with Meta-Analysis.

Supplementary Tables

Table S1. Data bases and search strategy used in the systematic review.

Database	Platform / Provider	Query Search	Limits Applied	Coverage	Results	Date of last search
MEDLINE	PubMed (NCBI)	(PICU[Title/Abstract] OR NICU[Title/Abstract] OR "Intensive Care Units, Pediatric"[MeSH Terms] OR "Intensive Care Units, Neonatal"[MeSH Terms]) AND (circadian*[Title/Abstract] OR "Circadian Rhythm"[MeSH Terms] OR sleep*[Title/Abstract] OR "Sleep"[MeSH Terms] OR melatonin[Title/Abstract] OR "Melatonin"[MeSH Terms] OR light[Title/Abstract] OR "Light"[MeSH Terms] OR noise[Title/Abstract] OR "Noise"[MeSH Terms]) NOT (animals[MeSH Terms] NOT humans[MeSH Terms])	No limits; Humans (via NOT animals)	From inception to search date	1,406	2025-10-01
ScienceDirect	Elsevier	('pediatric intensive care unit'/exp OR 'neonatal intensive care unit'/exp OR PICU:ti,ab,kw OR NICU:ti,ab,kw) AND	No limits; Humans	From inception to search date	1,819	2025-10-01

		('circadian rhythm'/exp OR 'sleep'/exp OR 'melatonin'/exp OR 'light'/exp OR 'noise'/exp)				
CINAHL	EBSCOhost	((MH "Intensive Care Units, Pediatric") OR (MH "Intensive Care Units, Neonatal") OR TI (PICU OR NICU) OR AB (PICU OR NICU) OR ((TI (intensive care OR "critical care") OR AB (intensive care OR "critical care")) N3 (TI (pediatric* OR paediatric* OR child* OR infant* OR neonat* OR newborn* OR adolescent* OR teen*) OR AB (pediatric* OR paediatric* OR child* OR infant* OR neonat* OR newborn* OR adolescent* OR teen*)))) AND ((MH "Circadian Rhythm") OR (MH "Chronotherapy") OR (MH "Sleep") OR (MH "Light") OR (MH "Lighting") OR (MH "Noise") OR (MH "Melatonin") OR TI (circadian* OR chronotherap* OR chronobiolog* OR sleep* OR melatonin OR light OR lighting OR illumination OR photoperiod* OR noise OR "quiet time" OR "dim light")	No limits	From inception to search date	1,962	2025-10-01

		OR AB (circadian* OR chronotherap* OR chronobiolog* OR sleep* OR melatonin OR light OR lighting OR illumination OR photoperiod* OR noise OR "quiet time" OR "dim light"))				
Web of Science Core Collection	Clarivate	TS=(((("intensive care" NEAR/3 pediatr*) OR ("intensive care" NEAR/3 child*) OR ("intensive care" NEAR/3 infant*) OR ("intensive care" NEAR/3 neonat*) OR ("intensive care" NEAR/3 newborn*) OR ("intensive care" NEAR/3 adolescen*) OR ("intensive care" NEAR/3 teen*) OR ("critical care" NEAR/3 pediatr*) OR ("critical care" NEAR/3 child*) OR ("critical care" NEAR/3 infant*) OR ("critical care" NEAR/3 neonat*) OR ("critical care" NEAR/3 newborn*) OR ("critical care" NEAR/3 adolescen*) OR ("critical care" NEAR/3 teen*) OR PICU OR NICU) AND (circadian* OR chronotherap* OR chronobiolog* OR sleep* OR melatonin* OR light OR lighting OR illumination OR photoperiod* OR noise	No limits	From inception to search date	1,991	2025-09-04

		OR "quiet time" OR "quiet hours" OR "dim light")))				
Scopus	Elsevier	TITLE-ABS-KEY(("pediatric intensive care" OR "neonatal intensive care" OR "pediatric critical care" OR PICU OR NICU) AND (circadian* OR chronotherap* OR chronobiolog* OR sleep* OR melatonin* OR light OR lighting OR illumination OR photoperiod* OR noise OR "quiet time" OR "dim light")))	No limits	From inception to search date	3,098	2025-10-01
LILACS	BVS	tw:(("unidades de cuidado intensivo neonatal" OR "neonatal intensive care units" OR "unidades de cuidado intensivo pediátrico" OR "pediatric intensive care units" OR "terapia intensiva neonatal" OR "terapia intensiva pediátrica" OR PICU OR NICU) AND ("ritmo circadiano" OR "circadian rhythm" OR cronoterapia OR chronotherapy OR sueño OR sleep OR melatonina OR melatonin OR luz OR lighting OR "iluminación" OR photoperiod* OR "fotoperiodo" OR ruido OR noise OR "tiempo de silencio" OR "quiet time"))	No limits	From inception to search date	187	2025-10-06
SciELO	BVS	(	No limits	From inception		

		("unidades de cuidado intensivo" OR "terapia intensiva" OR "intensive care" OR "critical care" OR PICU OR NICU)<br>AND (pediatr* OR paediatr* OR niñ* OR child* OR infant* OR neonat* OR newborn* OR adolescent*)<br>)<br>AND<br>(<br>"ritmo circadiano" OR "circadian rhythm" OR cronoterapia OR chronotherapy OR sueño OR sleep<br>OR melatonina OR melatonin OR luz OR lighting OR iluminación OR photoperiod* OR fotoperiodo<br>OR ruido OR noise OR "tiempo de silencio" OR "quiet time"<br>)		to search date	192	2025-10-06
Epistemonikos	Epistemonikos Foundation	(title:(circadian OR sleep OR melatonin OR light OR lighting OR illumination OR noise OR "quiet time" OR chronotherapy)<br>OR abstract:(circadian OR sleep OR melatonin OR light OR lighting OR illumination OR noise OR "quiet time" OR chronotherapy))<br>AND<br>(title:(NICU OR PICU OR "pediatric intensive care" OR "neonatal intensive care")<br>OR abstract:(NICU OR PICU OR "pediatric intensive care" OR "neonatal intensive care"))	No limits	From inception to search date	503	2025-10-06

ClinicalTrials.gov	NIH	(circadian OR sleep OR melatonin OR light OR lighting OR illumination OR noise OR "quiet time" OR chronotherapy) AND (pediatric OR paediatric OR child OR children OR infant OR neonatal OR neonate OR newborn) AND (ICU OR "intensive care" OR "critical care" OR PICU OR NICU)	No limits	All years	637	2025-10-06
WHO ICTRP	WHO	(circadian OR sleep OR melatonin OR light OR lighting OR illumination OR noise OR "quiet time" OR chronotherapy) AND (pediatric OR paediatric OR child OR children OR infant OR neonatal OR neonate OR newborn) AND (ICU OR "intensive care" OR "critical care" OR PICU OR NICU)	No limits	All years	104	2025-10-06
CENTRAL	Cochrane Library	#1 ICU = MeSH descriptor: [Intensive Care Units, Pediatric] explode all trees OR MeSH descriptor: [Intensive Care Units, Neonatal] explode all trees OR PICU:ti,ab,kw OR NICU:ti,ab,kw OR ("intensive care":ti,ab,kw OR "critical care":ti,ab,kw) #2 Circadian = MeSH descriptor: [Circadian Rhythm] explode all trees OR MeSH descriptor: [Sleep] explode all trees OR MeSH descriptor:	No limits	All years	0	2025-10-06

		[Melatonin] explode all trees OR MeSH descriptor: [Light] explode all trees OR MeSH descriptor: [Noise] explode all trees OR circadian*:ti,ab,kw OR sleep*:ti,ab,kw OR melatonin:ti,ab,kw OR (light OR lighting OR illumination OR photoperiod*):ti,ab,kw OR noise:ti,ab,kw OR "quiet time":ti,ab,kw Combine = #1 AND #2				

Table S2. Distribution of included studies according to the data synthesis strategy employed (Meta-Analysis vs. SWiM).

Group 1: Meta-Analysis (n=16)	Group 2: SWiM (n=16)	Group 3: Excluded from Synthesis (n=9)
Abdeyazdan et al. (2014)	AbouTurk et al. (2009)	Aita et al. (2013)
Akarsu et al. (2022)	Araújo et al. (2017)	Bazregari et al. (2019)
Düken & Yayan (2024)	Bloch-Salisbury et al. (2023)	Esmaeilizadeh et al. (2016)
Ezabadi et al. (2020)	Bradford et al. (2023)	Hamid et al. (2021)
Hendy et al. (2024)	Brandon et al. (2017)	Incekar et al. (2022)
Karami et al. (2018)	Curley et al. (2024)	Mony et al. (2018)
Khalesi et al. (2017)	Çetin & Ekici (2023)	Sánchez-Sánchez et al. (2022)
Lee et al. (2012)	Hellström-Westas et al. (2001)	Temizsoy et al. (2025)
Olgun et al. (2024)	Jung et al. (2005)	Vásquez-Ruiz et al. (2014)
Ray et al. (2025)	Kaneshi et al. (2016)	
Reyhani et al. (2014)	Mann et al. (1986)	
Suna Dağ et al. (2024)	Miller et al. (1995)	
Vadakkan et al. (2022)	Odebrecht et al. (2024)	
Valizadeh et al. (2017)	Rivkees et al. (2004)	
Zeraati et al. (2019)	Sabagh et al. (2024)	
Zhao et al. (2025)	Sato et al. (2018)	

* *Reason for Exclusion:* These studies reported outcomes that were incompatible with the meta-analysis domains (e.g., cortisol, weight velocity only) or lacked necessary variance data (SD).

Table S3. Pooled effect size by outcome

Outcome	# of studies (*K*)	N	SMD	95% CI	p-value	I^2	Effect Size
Heart Rate	8	542	-0.70	[-1.10,-0.30]	<0.001	79.6%	Large
Respiratory Rate	9	670	-0.75	[-1.16,-0.35]	<0.001	84.1%	Large
SpO2	7	474	+1.33	[0.95,1.72]	<0.001	71.7%	Very Large
Sleep Duration	4	256	+0.92	[0.66,1.19]	<0.001	0.0%	Large
